# Novel computational and drug design strategies for inhibition of monkeypox virus and *Babesia microti*: molecular docking, molecular dynamic simulation and drug design approach by natural compounds

**DOI:** 10.3389/fmicb.2023.1206816

**Published:** 2023-07-19

**Authors:** Shopnil Akash, Showkat Ahmad Mir, Sajjat Mahmood, Saddam Hossain, Md. Rezaul Islam, Nobendu Mukerjee, Binata Nayak, Hiba-Allah Nafidi, Yousef A. Bin Jardan, Amare Mekonnen, Mohammed Bourhia

**Affiliations:** ^1^Department of Pharmacy, Faculty of Allied Health Sciences, Daffodil International, University, Dhaka, Bangladesh; ^2^School of Life Sciences, Sambalpur University, Sambalpur, Odisha, India; ^3^Department of Microbiology, Jagannath University, Dhaka, Bangladesh; ^4^Department of Biomedical Engineering, Faculty of Engineering and Technology, Islamic University, Kushtia, Bangladesh; ^5^Department of Microbiology, West Bengal State University, Kolkata, West Bengal, India; ^6^Department of Food Science, Faculty of Agricultural and Food Sciences, Laval University, Quebec City, QC, Canada; ^7^Department of Pharmaceutics, College of Pharmacy, King Saud University, Riyadh, Saudi Arabia; ^8^Department of Biology, Bahir Dar University, Bahir Dar, Ethiopia; ^9^Department of Chemistry and Biochemistry, Faculty of Medicine and Pharmacy, Ibn Zohr University, Laayoune, Morocco

**Keywords:** molecular docking, molecular dynamics simulations, drug-likeness, admet, DFT, *Babesia microti*, monkeypox virus

## Abstract

**Background:**

The alarming increase in tick-borne pathogens such as human *Babesia microti* is an existential threat to global public health. It is a protozoan parasitic infection transmitted by numerous species of the genus Babesia. Second, monkeypox has recently emerged as a public health crisis, and the virus has spread around the world in the post-COVID-19 period with a very rapid transmission rate. These two novel pathogens are a new concern for human health globally and have become a significant obstacle to the development of modern medicine and the economy of the whole world. Currently, there are no approved drugs for the treatment of this disease. So, this research gap encourages us to find a potential inhibitor from a natural source.

**Methods and materials:**

In this study, a series of natural plant-based biomolecules were subjected to in-depth computational investigation to find the most potent inhibitors targeting major pathogenic proteins responsible for the diseases caused by these two pathogens.

**Results:**

Among them, most of the selected natural compounds are predicted to bind tightly to the targeted proteins that are crucial for the replication of these novel pathogens. Moreover, all the molecules have outstanding ADMET properties such as high aqueous solubility, a higher human gastrointestinal absorption rate, and a lack of any carcinogenic or hepatotoxic effects; most of them followed Lipinski’s rule. Finally, the stability of the compounds was determined by molecular dynamics simulations (MDs) for 100 ns. During MDs, we observed that the mentioned compounds have exceptional stability against selected pathogens.

**Conclusion:**

These advanced computational strategies reported that 11 lead compounds, including dieckol and amentoflavone, exhibited high potency, excellent drug-like properties, and no toxicity. These compounds demonstrated strong binding affinities to the target enzymes, especially dieckol, which displayed superior stability during molecular dynamics simulations. The MM/PBSA method confirmed the favorable binding energies of amentoflavone and dieckol. However, further *in vitro* and *in vivo* studies are necessary to validate their efficacy. Our research highlights the role of Dieckol and Amentoflavone as promising candidates for inhibiting both monkeypox and *Babesia microti*, demonstrating their multifaceted roles in the control of these pathogens.

## Graphical abstract

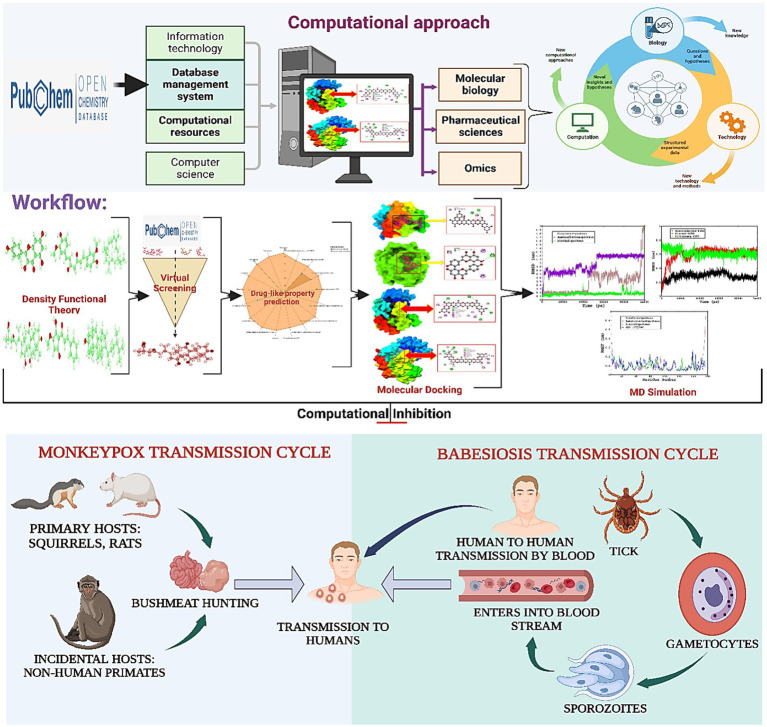

## 1. Introduction

Human babesiosis is a newly recognized tick-borne infection that is caused by an intraerythrocytic protozoan species belonging to the genus Babesia (Chand et al., 2022). In recent decades, the epidemiology of human babesiosis has shifted from a few isolated cases to the emergence of outbreaks in the northeastern and midwestern United States, and research has shown that human babesiosis develops within the red blood cells of humans and small rodents (Tanowitz and Weiss, 2009). There have been more than 100 human occurrences documented in the United States, with the most severe infections occurring in individuals who already have compromised immune systems (Djokic et al., 2018; Kumar et al., 2021). Another investigation suggested that *Babesia microti* is the most prominent causative agent of babesiosis in humans in the United States, especially in the Northeast and upper Midwest, where the disease is naturally occurring. Babesia parasites were first identified in 1888 in Romanian cattle (Vannier and Gelfand, 2020). The first human case of babesiosis was documented in the territory of the former Yugoslavia in 1957, and the second human case was reported in California in 1968 (Rosner et al., 1984). A year later, a third patient with babesiosis was discovered, and the species responsible for the disease was found to be *B. microti*. The patient was a native of Nantucket, which is located in the state of Massachusetts, and babesiosis was quickly identified as an epidemic ailment on the island (Kumar et al., 2021).

Human babesiosis can cause acute respiratory distress syndrome, hemolytic anemia, multiple organ failure, and death. While the parasite is transmitted to humans mainly by the bite of an infected tick, a growing number of instances of human-to-human transmission through blood transfusion have been documented (Mohr et al., 2000; Chiu et al., 2021).

More than one hundred species of Babesia have been reported, and these parasites can infect a wide variety of wild and domestic animals. Babesiosis is of major concern and pathogenicity, particularly in cattle, and has had a significant economic impact in several cattle-producing nations (Spielman, 1994; Kumar et al., 2021). *Babesia veratrum* is the principal species identified as a human pathogen. Several different genetically similar pathogen substrains have been documented to infect humans. These include the *Babesia divergens*-like and the *Babesia microti*-like viruses (Hunfeld et al., 2008; Kumar et al., 2021). According to the most recent statistics from the U.S. Centers for Disease Control and Prevention (CDC), more than 16,000 cases of babesiosis have been documented in the United States between 2011 and 2019, with the majority of confirmed cases in the Northeast. Most instances were recorded in New York, Massachusetts, and Connecticut during this period (Yannielli and Alcamo, 2009; U.S.NEWS, 2023). CDC researchers have described babesiosis as not endemic in Maine, New Hampshire, or Vermont, but these states have experienced increases comparable to or greater than those observed in areas where the infection is endemic (U.S.NEWS, 2023).

Furthermore, the viral zoonotic disease known as monkeypox is caused by the MPOX virus, which is related to the variola virus (which causes smallpox). Skin lesions or rashes that are typically limited to the face, hands, and feet are the hallmarks of the monkeypox infection. In 1970, a person in the Democratic Republic of the Congo was identified as the first human case of monkeypox. The subject was nine months old, and the incident occurred in a region of the country where smallpox had been eradicated as recently as 1968. Since then, the vast majority of reports have come from remote, tropical areas of the Congo Basin, primarily in the Democratic Republic of the Congo, with increasing evidence that the disease is spreading throughout Central and West Africa (Parker et al., 2007; Farasani, 2022). In 1970, clinical isolates of monkeypox were found in 11 African countries, namely Benin, Cameroon, the Central African Republic, the Democratic Republic of the Congo, Gabon, Liberia, Nigeria, Sierra Leone, and South Sudan (Beer and Rao, 2019). The potential severity of monkeypox in humans is still unknown. For example, in 1996 and 1997, the Democratic Republic of the Congo experienced an outbreak with unusually high incidence rates but a lower-than-usual case fatality rate. Since 2017, more than 500 new cases, 200 of which were confirmed, and a case fatality rate of approximately 3% have all been reported in Nigeria (Yinka-Ogunleye et al., 2018; Antunes et al., 2022). Thus, monkeypox is currently considered a threat not only to countries in West and Central Africa but to the entire world. This means that it is a significant public health concern worldwide.

In 2003, the United States of America became the first country outside of Africa to experience an outbreak of monkeypox (Sah et al., 2022). Prairie dogs kept as pets were identified as the source of this infection. These pets shared a cage with dormice and Gambian pouched rats illegally imported from Ghana. More than 70 cases of monkeypox have been identified in the United States as a result of this epidemic (Kabuga and El Zowalaty, 2019). Travelers from Nigeria have also been reported to have developed monkeypox in Israel in September 2018, in the United Kingdom in September 2018, in Singapore in December 2019, May 2021, and May 2022 (Adegboye et al., 2022), and in the United States of America in July and November 2021 (Kumar et al., 2022). In May 2022, multiple occurrences of monkeypox were detected in different countries. Since the beginning of May 2022, more than 3,000 cases of monkeypox virus infection have been documented in approximately 50 countries in five regions. These findings prompted the World Health Organization (WHO) to declare monkeypox an “emerging global public concern” on 23 June 2022 (Thornhill et al., 2022).

Although human babesiosis and monkeypox are both life-threatening conditions, there are currently no effective therapies or vaccines available to combat these diseases. Therefore, a viable drug to control human babesiosis and the monkeypox infection is urgently required to prevent another pandemic like SAR CoV-2 (Kumar et al., 2022; Nolasco et al., 2022). Thus, this study intends to explore potentially valuable drugs derived from natural sources. Since nature is regarded as a fantastic source of cures for all kinds of diseases (Jafari Porzani et al., 2022), in this case, *in silico* methods were chosen, and different drug design approaches were applied to establish them as potential candidates.

*In silico* strategies provide a framework for assessing the function of potential therapeutics against specific biological targets, which enables the selection of those with the best possible drug candidate for further *in vitro* and *in vivo* studies (Vougas et al., 2019). *In silico* techniques can also be used to monitor the function of existing therapeutics against biological targets. These techniques have reduced the time and cost of novel drug development by minimizing the use of resources in laboratory testing (Yu et al., 2022). The investigation of the effectiveness of natural product-derived drugs against monkeypox and MERS CoV-2 viral proteins is one of the areas where *in silico* approaches are beneficial. In the field of biomedicine, the development of *in silico* assays has proven to be quite beneficial, including molecular docking, molecular dynamics simulation, and ADMET analysis, which are both straightforward and reliable. Thus, this innovative research was conducted to investigate the efficacy of natural inhibitors against the monkeypox virus and *B. microti* (Tabti et al., 2022).

## 2. Materials and methods

### 2.1. Ligand preparation and molecular optimization

**Figure 1:**
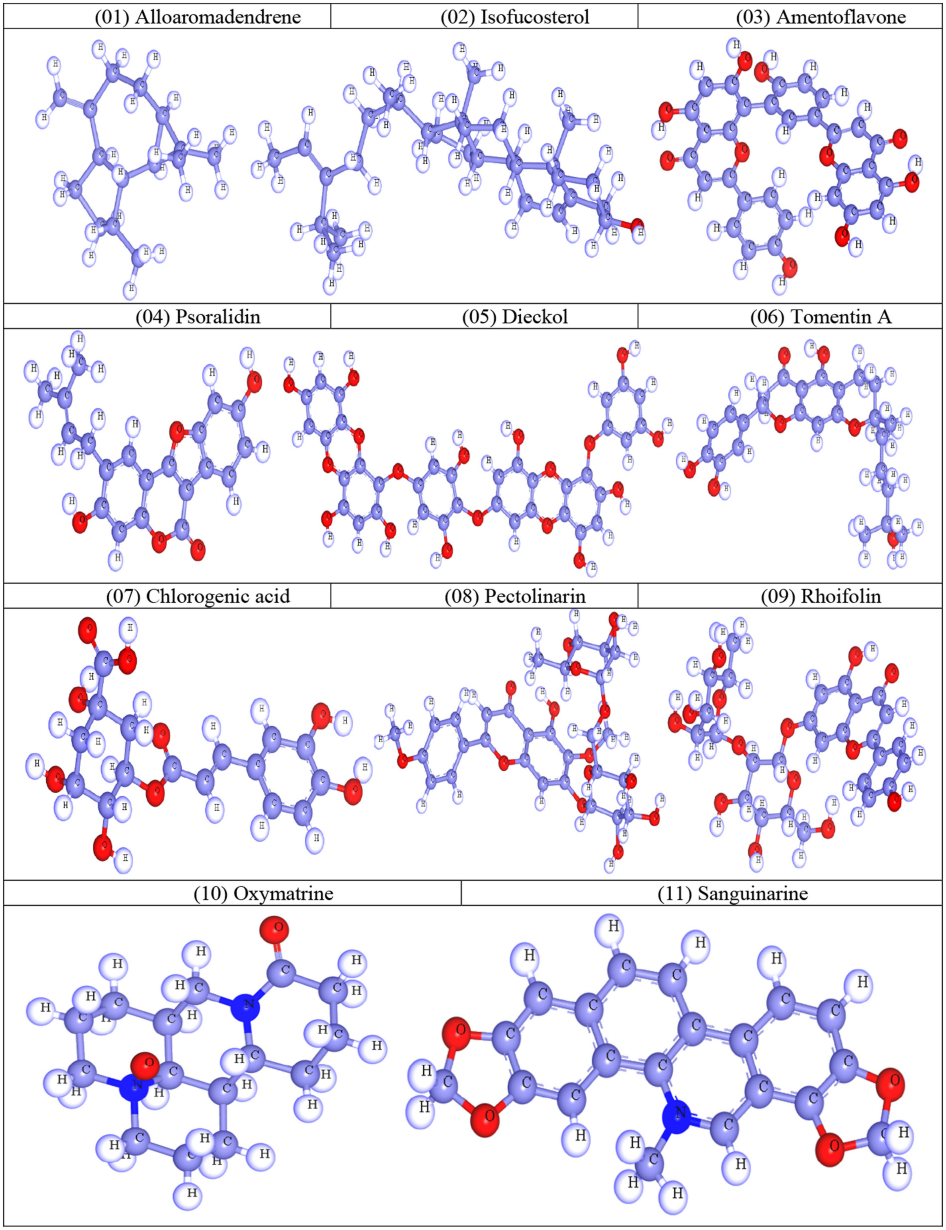
Optimized molecular structures of natural compounds.

Calculation of thermodynamic, molecular orbital, and molecular electrostatic characteristics is essential to computational chemistry, and quantum mechanical techniques are often used in this field. Gaussian 09 software was used to refine and optimize the geometry of selected natural molecules (Gaussian et al., 2009). Then, the optimization process was carried out using DFT (3-21G) with Becke’s and Lee, Yang, and Parr’s (LYP) (B) functional theory. During the optimization process, water was used as the solvent medium. After optimization, all compounds were saved in SDF format for further computational work (Amin et al., 2022). Finally, the optimized structures were viewed in Material Studio 08, and the 3D structure was captured as shown in Figure 1.

### 2.2. Protein preparation and molecular docking study and visualization

**Figure 2:**
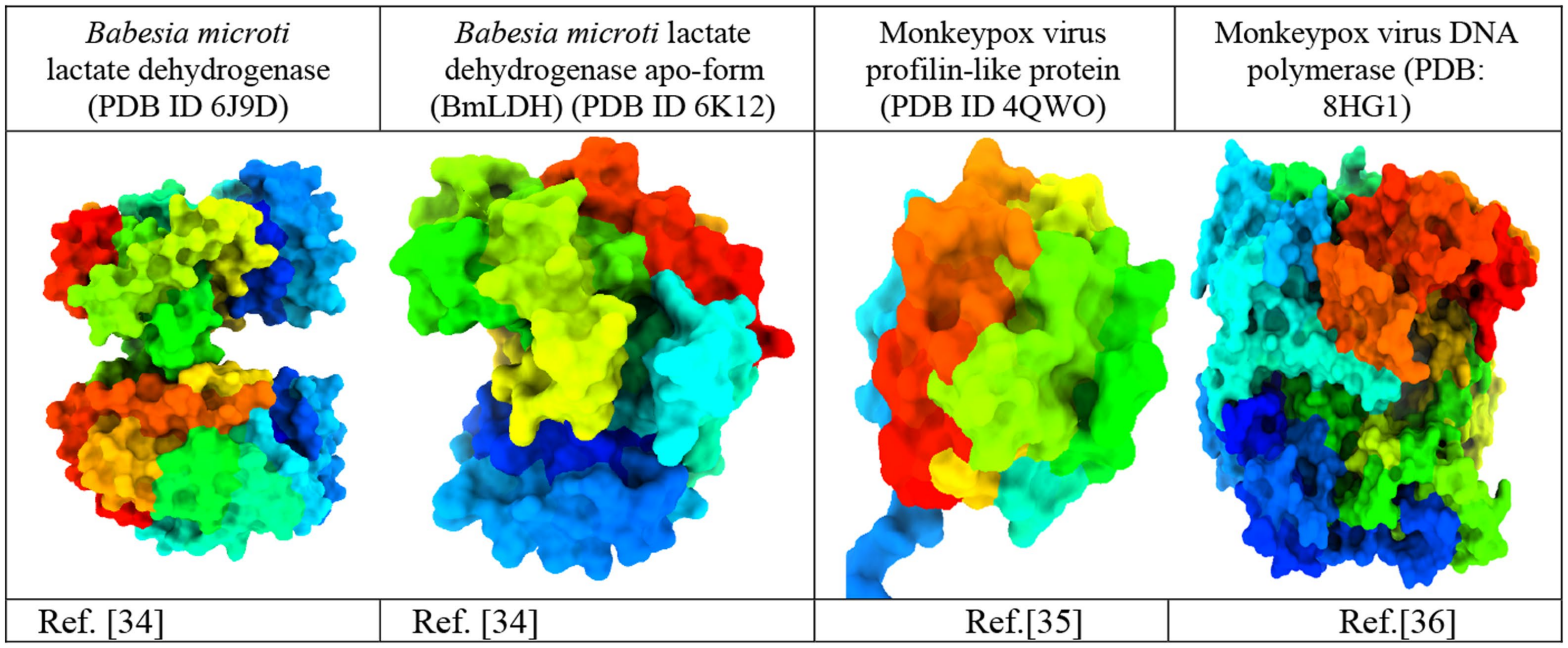
Three-dimensional protein structure of the *monkeypox virus* (Minasov et al., 2014; Yu et al., 2019; Peng et al., 2022).

The crystal structures of *Babesia microti* lactate dehydrogenase (PDB ID 6J9D), *Babesia microti* lactate dehydrogenase apo-form (PDB ID 6 K12), *monkeypox virus profilin-like protein (PDB ID 4QWO),* and monkeypox virus DNA polymerase (PDB: 8HG1) were acquired from the RCSB Protein Data Bank (https://www.rcsb.org/; Burley et al., 2017). The three-dimensional protein structures were imported into the BIOVIA Discovery Studio Visualizer software to remove water molecules and unwanted heteroatoms (Figure 2). Water molecules often have no role in the substrate’s ability to bind to the receptor. They were thus removed to accelerate computations and free up the binding site. Additionally, the intended active site of the receptor may be occupied by previously docked, unwanted heteroatoms. As a consequence, they were removed to free up the active site and speed up computations. Swiss PDB Viewer v.4.10 was employed for energy minimization (Sharma et al., 2019). PyRx version 0.8 virtual screening tools were used for docking with AutoDock Vina (Dallakyan and Olson, 2015). Finally, the docking results, complex structure, binding affinity, non-binding interactions, and binding pocket were visualized using the BIOVIA Discovery Studio Visualizer 4.5 software tools (Nath et al., 2021).

### 2.3. Determination of ADMET, Lipinski’s rule, and pharmacokinetics

Drug-like and non-drug-like compounds were differentiated using Lipinski’s rule of five. Drug-like criteria were used to more efficiently determine a molecule’s drug-like qualities in its structural characteristics (Walters et al., 1999; Walters and Murcko, 2002). Important pharmacokinetic properties such as hydrogen bond acceptor, hydrogen bond donor, TPSA, bioavailability, molecular weight, and consensus log Po/w were estimated using SwissADME (http://www.swissadme.ch/index.php; Daina et al., 2017). Lipinski’s Rule was also determined using the same web server.
ADMET stands for Absorption, Distribution, Metabolism, Excretion, and Toxicity. These pharmacological properties are determined for each drug candidate. Drug development significantly depends on ADMET characteristics, with 50% of drugs failing because they violate these pharmacokinetic principles (Li, 2001). *In silico* ADMET studies were performed using an online web tool server called pkCSM (https://biosig.lab.uq.edu.au/pkcsm/prediction; Pires et al., 2015). Several pharmacokinetic parameters such as water solubility, coca-2 permeability, human intestinal absorption, Blood–Brain Barrier (BBB) penetration, cytochrome P450 inhibition and substrate, AMES toxicity, skin sensitization, and hepatotoxicity levels were calculated for selected natural compounds.

### 2.4. Calculation of QSAR and pIC_50_

The abbreviation of QSAR is Quantitative Structure–Activity Relationship, which establishes a relationship between the chemical structure and the biological activity of chemical compounds (Miladiyah et al., 2018). QSAR is a quantum chemistry method that predicts the efficacy of compounds in drug discovery and development (Devillers, 1996). To perform QSAR and pIC_50_ we used a freely available website called ChemDes (http://www.scbdd.com/chemopy_desc/index/; Kumer et al., 2019). These web services provided data such as Chiv5 molecular connectivity, bcutm1 mean burden descriptors, MRVSA9, MRVSA6, and PEOEVSA5 are MOE type descriptors and GATSv4 indicating autocorrelation descriptors, with the last two parameters J and diameter suggesting topological descriptors of drug molecules for reported ligands. To determine and calculate the QSAR and pIC_50,_ the mentioned parameters were first collected from the ChemDes database, and after developing the multiple linear regression (MLR) in an Excel sheet and calculating the pIC_50_ value, the mentioned MLR equations were applied for calculating the pIC50 values.

pIC50 (Activity) = −2.768483965 + 0.133928895 × (Chiv5) + 1.59986423 × (bcutm1) + (− 0.02309681) × (MRVSA9) + (− 0.002946101) × (MRVSA6) + (0.00671218) × (PEOEVSA5) + (− 0.15963415) × (GATSv4) + (0.207949857) × (J) + (0.082568569) × (Diametert) (Siddikey et al., 2022).

### 2.5. Molecular dynamics simulations

Molecular dynamics simulations were performed using the GROMACS package on the docked protein-ligand complex to determine structural stability and protein properties. The simulation was carried out for 100 ns in water, and the AMBER force field was used. Trajectory and energy files were noted every 2 fs (Mir et al., 2022).

For solvation purposes, we used truncated cubic boxes that contained TIP3P water molecules. The box dimensions and vectors were set to 3.256 × 3.061 × 3.142 nm and 5.7 × 5.7 × 5.7 nm, respectively. To effectively comply with the minimum image convention, the protein was centered in the simulation box at a minimum distance of 1 nm from the box edge. The entire system contained 56,242 atoms, and the simulation was executed in 0.15 M KCL by adding the necessary potassium and chloride ions. Energy minimization is a critical step to avoid static clashes. Therefore, energy minimization was performed using the steepest descent method for 5,000 steps. The process was terminated when the total maximum force of the system reached <1,000 (KJ mol − ^1^ nm − ^1^), followed by a brief 100 ps (50,000 steps) equilibration in the NVT ensemble and then 1,000 ps (1,000,000 steps) in the NPT ensemble.

Furthermore, a stable temperature and pressure of 300 K and 1 atm were maintained using the Parrinello-Rahman algorithm for weak coupling velocity rescaling (modified Berendsen thermostat). The relaxation intervals were set to τ T = 0.2 ps and τ *p* = 1.0 ps. A Verlet scheme was applied to calculate non-bonded interactions. All x, y, and z directions used Periodic Boundary Conditions (PBC). Interactions within a short-range threshold of 1.2 nm were calculated at each time step. The electrostatic interactions and forces for a homogeneous medium outside the long-range limit were calculated using Particle Mesh Ewald (PME). The trajectories generated during the 100 ns production run were utilized to calculate the radius of gyration (Rg), Root-Mean Square Deviation (RMSD), and Root-Mean Square Fluctuation (RMSF). All graphs were generated and visualized in Xmgrace.

### 2.6. Free energy calculations

The free binding was calculated using the traditional MM/PBSA method. The trajectories generated during the MD simulations were used for the free binding energy calculations. The ionic strength of the system was 0.150 M concentration with default grid dimensions. The non-polar solvation energy was calculated using the solvent-accessible surface area (SASA) model. The default values of solvent surface tension and SASA energy constant from previous MM/PBSA calculations of 0.0226778 kJ/mol Å^2^ and 3.84982 kJ/mol, respectively, were also used. The average free binding energy of dieckol, amentoflavone, and the co-crystallized ligand cidofovir with monkeypox virus profilin-like protein and monkeypox virus DNA polymerase was determined using the MM/PBSA method (El-Barghouthi et al., 2009). The average free binding energy was calculated by the bootstrap method using MmPbSaStat.py. The decomposition energy of each amino acid contributing to the free energy binding was calculated using the MmPbSaDecomp.py module of *g_mmpbsa*. The ΔG_bind_ was calculated according to the following equation (1).


(1) $$\;{\mathrm{\Delta G}}_{\mathrm{binding}}=\Delta G_{\mathrm{complex}}-\left(\Delta G_{\mathrm{protein}}\right)+\left(\Delta G_{\mathrm{ligand}}\right)$$

The above-mentioned method has been implemented by several authors to calculate the free binding energy of molecular scaffolds with template targets (Mir and Nayak, 2021; Mir et al., 2022, 2023).

### 2.7. DFT calculation

Optimizations of the selected phytochemicals were performed by use of functional B3LYP and basis set 6-311G++ of Gaussian 09v program (Kashyap et al., 2021; Kumer et al., 2022). The electronegative atom, oxygen, was assumed to be common to produce accurate results. Once the geometric optimization was done, molecular frontier orbital diagrams were identified: HOMO and LUMO. The HOMO and LUMO orbitals and their corresponding magnitudes were created using vibrational frequencies from the visualization interface of the Gaussian 09 program. The LUMO and HOMO were calculated from these frontier orbital energies, and the energy gap (E gap) was determined. Finally, the hardness (η), and softness (S) were observed through the DFT approach, which refers to the behavior of the molecule.

## 3. Analysis of the results

### 3.1. Lipinski’s rule and pharmacokinetics

The drug-like assessment is a qualitative approach used to develop drugs or drug-like substances with respect to various parameters, such as bioavailability. In addition, the field of pharmacokinetics describes what happens to a chemical after it is absorbed by a living organism. Drug-likeness methods and Lipinski’s rule of five help predict pharmacokinetic parameters based on the structure of the compound (Lipinski, 2004). The majority of the selected compounds in the current study, excluding dieckol, pectolinarin, and rhoifolin adheres to Lipinski’s rule of five. These three molecules obeyed Lipinski’s rule of five due to their higher molecular weights. Therefore, by ignoring the molecular weight, we continued with further computational studies. All the drug compounds in Table 1 have good bioavailability scores (most of them 0.55, or 55%), which indicate how much of the medication is absorbed into the bloodstream—lipophilicity, expressed by the logarithm of the octanol–water partition coefficient log P. In the development of new drugs, the assessment of lipophilicity values should be significant to understand the affinity of any drug to a lipid environment (Pallicer et al., 2014).

**Table 1:** Lipinski’s rule of five data, pharmacokinetics.

PubChem CID	Ligand name	Molecular weight	Hydrogen bond acceptor	Hydrogen bond donor	Consensus log P_o/w_	Lipinski’s rule		Bioavailability
						Result	Violation	
91,354	Alloaromadendrene	204.35	0	0	4.34	Yes	1	0.55
5,281,326	Isofucosterol	412.69	1	1	7.07	Yes	1	0.55
5,281,600	Amentoflavone	538.46	10	6	3.62	No	2	0.17
5,281,806	Psoralidin	336.34	5	2	3.98	Yes	0	0.55
3,008,868	Dieckol	742.55	18	11	3.39	No	3	0.17
71,659,627	Tomentin A	442.5	7	4	3.59	Yes	0	0.55
1,794,427	Chlorogenic acid	354.31	9	6	−0.38	Yes	1	0.11
168,849	Pectolinarin	622.57	15	7	−0.43	No	3	0.17
5,282,150	Rhoifolin	578.52	14	8	−0.66	No	3	0.17
114,850	Oxymatrine	264.36	2	0	0.41	Yes	0	0.55
5,154	Sanguinarine	332.33	4	0	2.88	Yes	0	0.55

### 3.2. Molecular docking analysis against the targeted receptor of the monkeypox virus

The molecular docking technique predicts the binding orientation of a ligand molecule to a specific biological macromolecule. This approach is also constructive for assessing how drug molecules can bind to biological target (Singh et al., 2022). Monkeypox virus profilin-like protein and monkeypox virus DNA polymerase were selected as receptor molecules to perform molecular docking. Blind docking was included for both receptors (Table 2). Dieckol and amentoflavone exhibited the most promising docking outcomes, with dieckol having a binding affinity of −10.1 Kcal/mol for profilin-like protein and − 10.5 Kcal/mol for monkeypox virus DNA polymerase. This suggests that dieckol forms a stronger bond with the receptor macromolecules than cidofovir (the standard drug) and that this compound may play a critical role in the long-term stabilization of this protein. On the other hand, amentoflavone also showed strong binding affinity where the compound binds with profilin-like protein*s* with −9.5 Kcal/mol and amentoflavone-monkeypox DNA polymerase-bound complex exhibited binding affinity of −10.3 Kcal/mol. The docking profile for the rest of the compounds (except alloaromadendrene) was also promising, as these compounds also exhibited better binding affinity than cidofovir, but dieckol and amentoflavone were selected for further investigation with the most convincing binding affinity.

**Table 2:** Binding affinity against the *monkeypox* virus.

Name of compound	Monkeypox virus profilin-like protein (PDB ID: 4QWO)	Monkeypox virus DNA polymerase (PDB: 8HG1)
	Binding affinity (Kcal/mol)	Binding affinity (Kcal/mol)
Alloaromadendrene	−6.3	−6.9
Isofucosterol	−8.3	−8.7
Amentoflavone	−9.5	−10.3
Psoralidin	−8.6	−8.5
Dieckol	−10.1	−10.5
Tomentin A	−9.1	−9.1
Chlorogenic acid	−8.0	−6.9
Pectolinarin	−8.4	−9.2
Rhoifolin	−8.5	−9.6
Oxymatrine	−7.0	−7.3
Sanguinarine	−8.3	−9.0
Standard Cidofovir	−6.5	−6.8

### 3.3. Molecular docking analysis against *targeted* human *Babesia microti*

*Babesia microti* lactate dehydrogenase and *Babesia microti* lactate dehydrogenase apo-form were docked against ligands selected to determine which ligands should be considered for molecular dynamics simulations. For *Babesia microti* lactate dehydrogenase, the standard drug diminazene expressed a binding affinity of −6.4 Kcal/mol. All the selected ligands showed better binding affinity than the standard drug, with amentoflavone (−10.2 Kcal/mol) and dieckol (−11.1 Kcal/mol) exhibiting the strongest binding affinity for the receptor. On the other hand, diminazene had a binding affinity of −6.2 Kcal/mol for the apo-form of *Babesia microti* lactate dehydrogenase. Amentoflavone (−10.0 Kcal/mol) and dieckol (−10.4 Kcal/mol) again expressed robust binding affinity toward receptors. Isofucosterol, psoralidin, tomentin A, chlorogenic acid, pectolinarin, rhoifolin, oxymatrine, and sanguinarine also exhibited promising docking scores (Table 3) against both receptors, with outstanding binding affinity but lower than amentoflavone and dieckol.

**Table 3:** Binding affinity against human *Babesia microti.*

Name	*Babesia microti* lactate dehydrogenase (PDB ID: 6J9D)	*Babesia microti* lactate dehydrogenase apo form (PDB ID: 6 K12)
	Binding affinity (Kcal/mol)	Binding affinity (Kcal/mol)
Alloaromadendrene	−6.5	−6.2
Isofucosterol	−7.9	−9.1
Amentoflavone	−10.2	−10.0
Psoralidin	−8.2	−7.9
Dieckol	−11.1	−10.4
Tomentin A	−8.1	−8.2
Chlorogenic acid	−7.3	−7.5
Pectolinarin	−8.2	−8.9
Rhoifolin	−8.7	−8.7
Oxymatrine	−7.5	−6.8
Sanguinarine	−9.2	−8.4
Standard Diminaze	−6.4	−6.2

### 3.4. Molecular docking pose and interaction analysis

**Figure 3:**
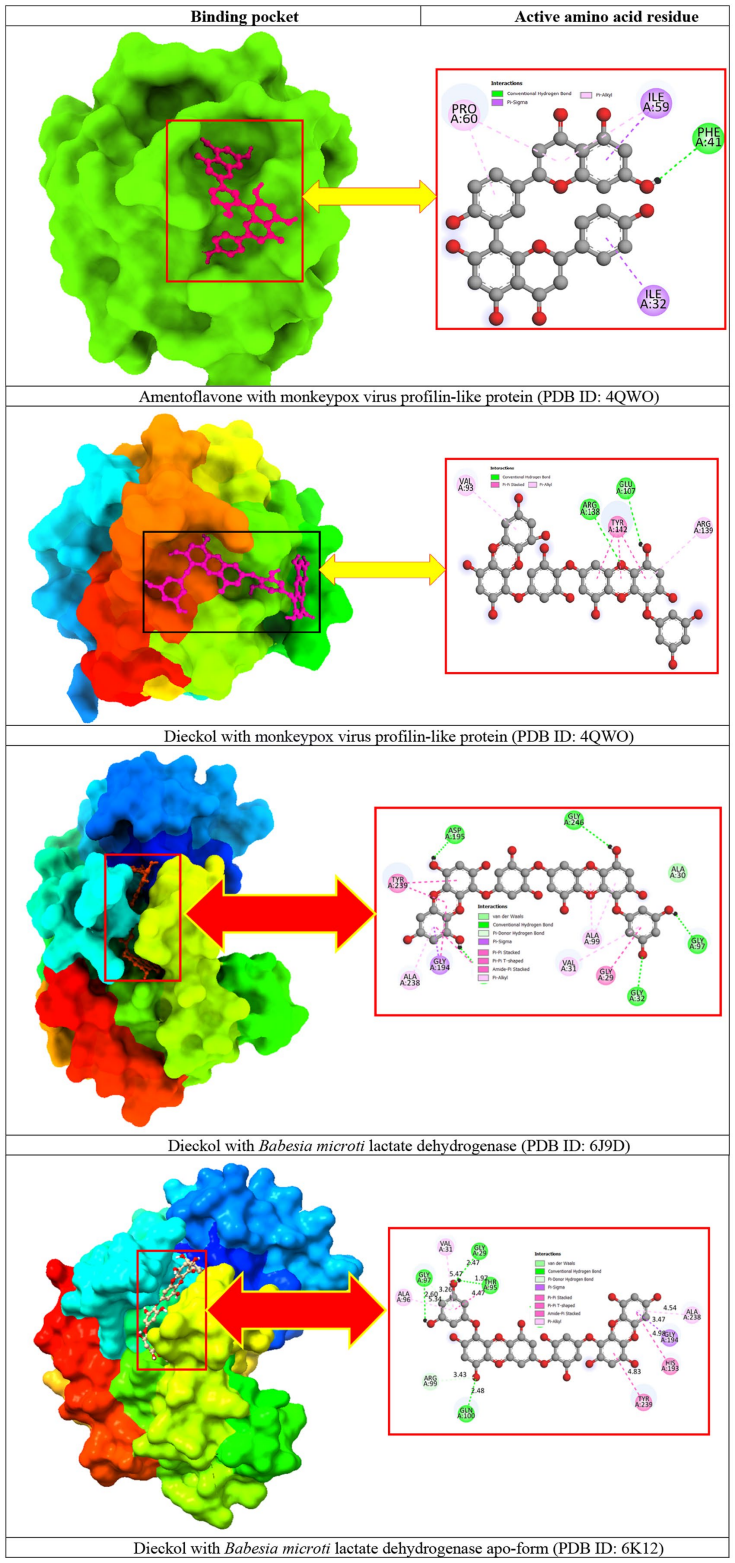
Docking interactions between the proposed compounds.

Molecular docking allows the prediction or investigation of the interactions that are the key components that substantially influence the affinity of a ligand for a receptor. According to molecular docking studies, the receptor-ligand complex with the lowest amount of energy excreted is considered to have the highest binding affinity. From the previous step, we selected amentoflavone and dieckol as the most suitable ligands with the lowest binding energy and the highest binding affinity score for selected proteins. The receptor protein-ligand docked complexes are shown in Figure 3.

For *the monkeypox virus profilin-like protein,* amentoflavone formed a pi-sigma bond with ILE A:32 and ILE A:59; a pi-alkyl bond with PRO A:60; a conventional hydrogen bond with PHE A:41. On the other hand, dieckol formed a conventional hydrogen bond with ARG A:138 and GLU A:107; a pi-alkyl bond with VAL A:93 and ARG A:139; a pi-pi stacking with TYR A:142. There was no pi-sigma bond observed for dieckol.

Additionally, for *Babesia microti* lactate dehydrogenase, dieckol formed a conventional hydrogen bond with GLY A:32, GLY A:97, ASP A:195, and GLY A:246; a pi-alkyl bond with VAL A:31, ALA A:99, and ALA A:238; a Van der Waals bond with ALA A:30; a pi-sigma bond with GLY A:194; a pi-pi stacking with GLY A:29; a pi-pi T-shaped with TYR A:239. On top of that, dieckol with *Babesia microti* lactate dehydrogenase apo-form formed a conventional hydrogen bond with GLY A:29, GLY A:97, THR A:95, and GLN A:100; a pi-alkyl bond with VAL A:31, ALA A:96, and ALA A:238; a pi-sigma bond with GLY A:194; a pi-pi stacking with HIS A:193 and TYR A:239, and a pi-donor hydrogen bond was observed at residue ARG A:99.

### 3.5. ADMET data analysis

The pharmacokinetic properties of the studied compounds are shown in Table 4. We evaluated the absorption features of the compounds based on water solubility, Caco-2 permeability, and human intestinal absorption. Drug absorption depends on water solubility; a higher water-soluble compound implies higher absorption properties and ample bioavailability (Xu et al., 2013). Compound 02 is more water-soluble than compound 07, which is marginally more water-soluble. Caco-2 is a human colorectal adenocarcinoma cell line that has been immortalized and is primarily utilized as a reference model of the intestinal barrier (Lea, 2015). Compounds 11 and 05 show maximum (2.107) and minimum Caco-2 permeability (−0.967). The human intestinal absorption (HIA) rate is essential in predicting how well a medicine will be absorbed when administered orally (Arora and Khurana, 2022). Compound 09 has the lowest percentage (24.308%) of HIA, while compound 11 has the maximum percentage (100%) of Hof IA. Table 4 displays drug distribution characteristics such as the blood–brain barrier and volume of distribution (VD). A lower VD score (<−0.15) indicates that the therapeutic agent is more uniformly distributed in plasma as opposed to tissue, whereas a higher VD score (>0.45) reflects that the pharmaceutical molecule is more uniformly transported in tissues. Compound 03 has the lowest VD value, but compound 09 has a higher VD value. Additionally, the blood–brain barrier (BBB) prevents the substance from entering the brain and central nervous system. Only two of our reported biomolecules, alloaromadendrene, and isofucosterol, can cross the BBB.

**Table 4:** Thermotical ADME properties.

**No**	**Water solubility Log S**	**Caco-2 permeability x 10** ^**−6**^	**Human intestinal absorption (%)**	**VDss (human)**	**BBB permeability**	**CYP450 1A2 inhibitor**	**CYP450 2D6 substrate**	**Renal OCT2 substrate**	**AMES toxicity**	**Skin sensitization**	**Hepatotoxicity**
01	−5.764	1.395	95.302	0.753	Yes	No	No	No	No	No	No
02	−6.715	1.212	94.642	0.179	Yes	No	No	No	No	No	No
03	−2.892	0.145	84.356	−1.066	No	No	No	No	No	No	No
04	−3.979	1.048	93.488	0.052	No	Yes	No	No	Yes	No	No
05	−2.892	−0.967	68.892	−0.218	No	No	No	No	No	No	No
06	−3.502	−0.366	86.57	0.615	No	No	No	No	No	No	No
07	−2.449	−0.84	36.377	0.518	No	No	No	No	No	No	No
08	−2.986	0.309	41.847	0.684	No	No	No	No	No	No	No
09	−2.862	−0.942	24.308	1.14	No	No	No	No	No	No	No
10	−3.58	1.269	96.121	0.404	No	No	No	No	No	No	No
11	−5.56	2.107	100.	0.298	No	Yes	No	No	Yes	No	No

Following drug distribution, the liver breaks down the compound through various enzymatic processes. The isoenzyme cytochrome P450 is in charge of the biotransformation and metabolism of drugs (Cresteil et al., 1994). The importance of drug metabolism by cytochrome P450 can be attributed to concerns about medication toxicity and pharmacological effects (Ogu and Maxa, 2000). There are several routes for a drug molecule to be excreted from the body, namely through the liver, bile, and kidneys. A valuable piece of information for estimating drug excretion is the total clearance rate of the drug molecule. It indicates the amount of drug excreted per unit by the combination of the liver and kidney (Dowd et al., 2010). Organic cation transporter two, or OCT2 substrate, is a crucial excretion factor because it improves renal clearance. None of the compounds were predicted to act as OCT2 substrates. One of the leading causes of unsuccessful medication development is toxicity. None of our compounds showed any toxicity, such as skin sensitivity or liver damage, except compounds 04 and 11, which showed AMES toxicity.

### 3.6. Quantitative structure–activity relationship and pIC_50_

The Quantitative Structure–Activity Relationship (QSAR) is a computer modeling approach that has been applied and used in the area of drug discovery and design to predict the biological activity of chemical compounds based on their molecular structures. This is accomplished and used mainly in the development of new drugs, especially in computer-aided drug design. It involves the development of mathematical models that connect the physicochemical descriptions or structural aspects of molecules to their biological action (Patel et al., 2014; Wang et al., 2015).

The standard ranges of QSAR are considered below 10. Any molecule below 10 is potential, according to the theory (Ahamed et al., 2023). In our current investigation, the overall values of QSAR and pIC_50_ are positive (Table 5) and they are satisfied with standard ranges. The highest and lowest values of pIC_50_ are 6.26 and 4.45, respectively. The outcome of PIC_50_ suggests that the compound may have therapeutic efficacy against the targeted disease.

**Table 5:** Data of QSAR calculation.

Ligand	Chiv5	bcutm1	(MRVSA9)	(MRVSA6)	(PEOEVSA5)	GATSv4	J	Diameter	PIC50
01	3.846	3.972	0.0	12.52	32.923	0.0	1.898	6.0	5.18
02	6.776	3.972	0.0	23.298	57.917	0.566	1.447	15	6.26
03	3.214	4.156	21.938	93.243	0.0	0.878	1.195	17	5.04
04	2.199	4.247	32.908	57.965	11.649	0.951	1.493	12	4.62
05	3.68	4.04	0.0	66.73	0.0	0.809	0.873	25	6.11
06	3.192	4.001	5.783	40.956	6.066	0.834	1.395	16	5.32
07	1.708	3.866	18.015	29.839	6.066	1.172	1.829	13	4.45
08	3.365	4.116	10.969	46.622	0.0	0.957	1.236	21	5.71
09	3.236	4.106	10.969	52.688	0.0	0.961	1.286	18	5.43
10	4.694	3.931	5.907	5.207	0.0	1.002	1.676	7	4.76
11	2.846	4.215	32.448	42.595	6.066	0.761	1.27	11	4.57

### 3.7. Molecular dynamics simulations for monkeypox complexes

**Figure 4:**
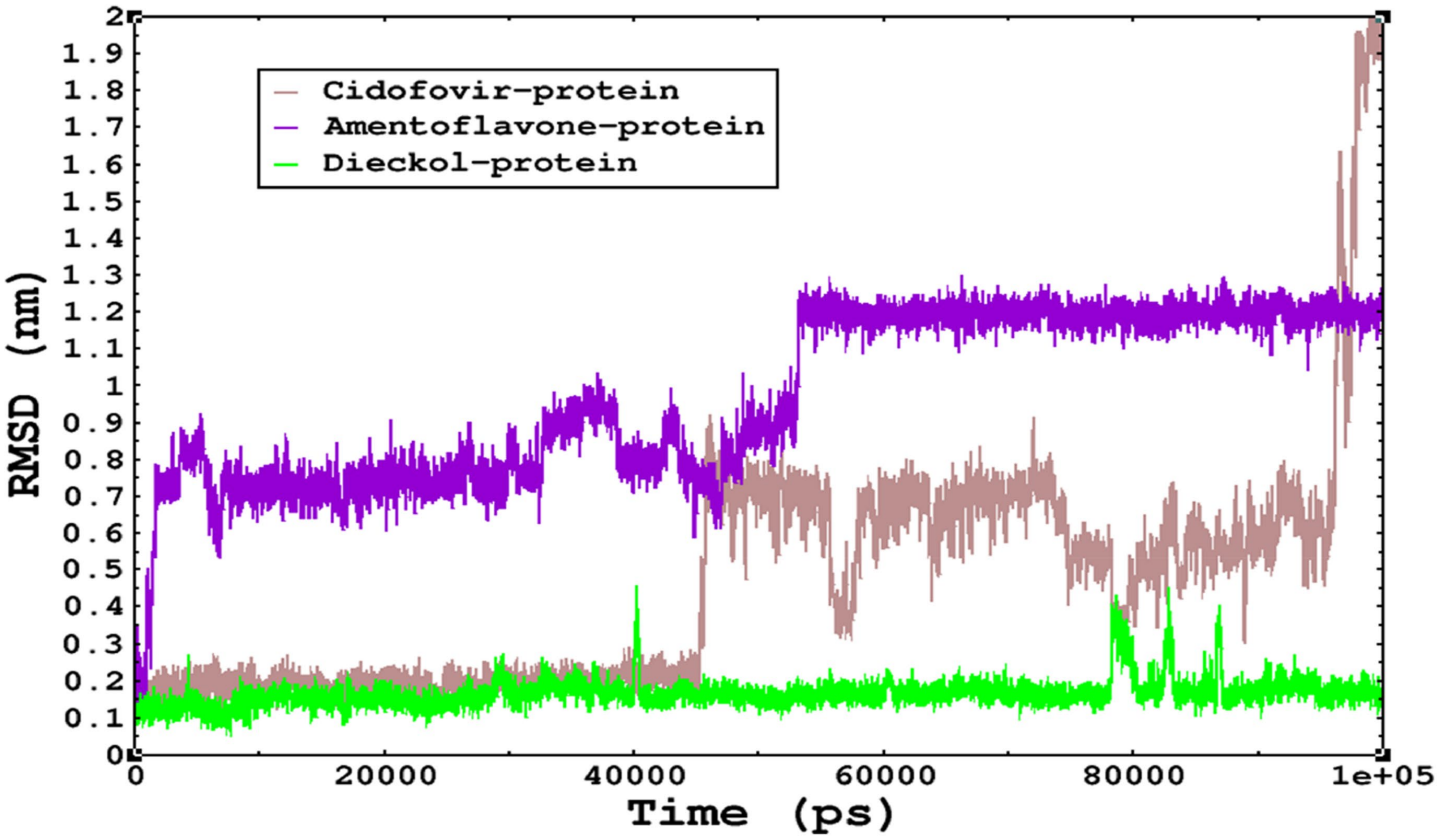
The conformations of dieckol, cidofovir, and amentoflavone with *monkeypox* virus during simulations in an aqueous medium. The trajectories are plotted, and the color represented is given in the legend box.

**Figure 5:**
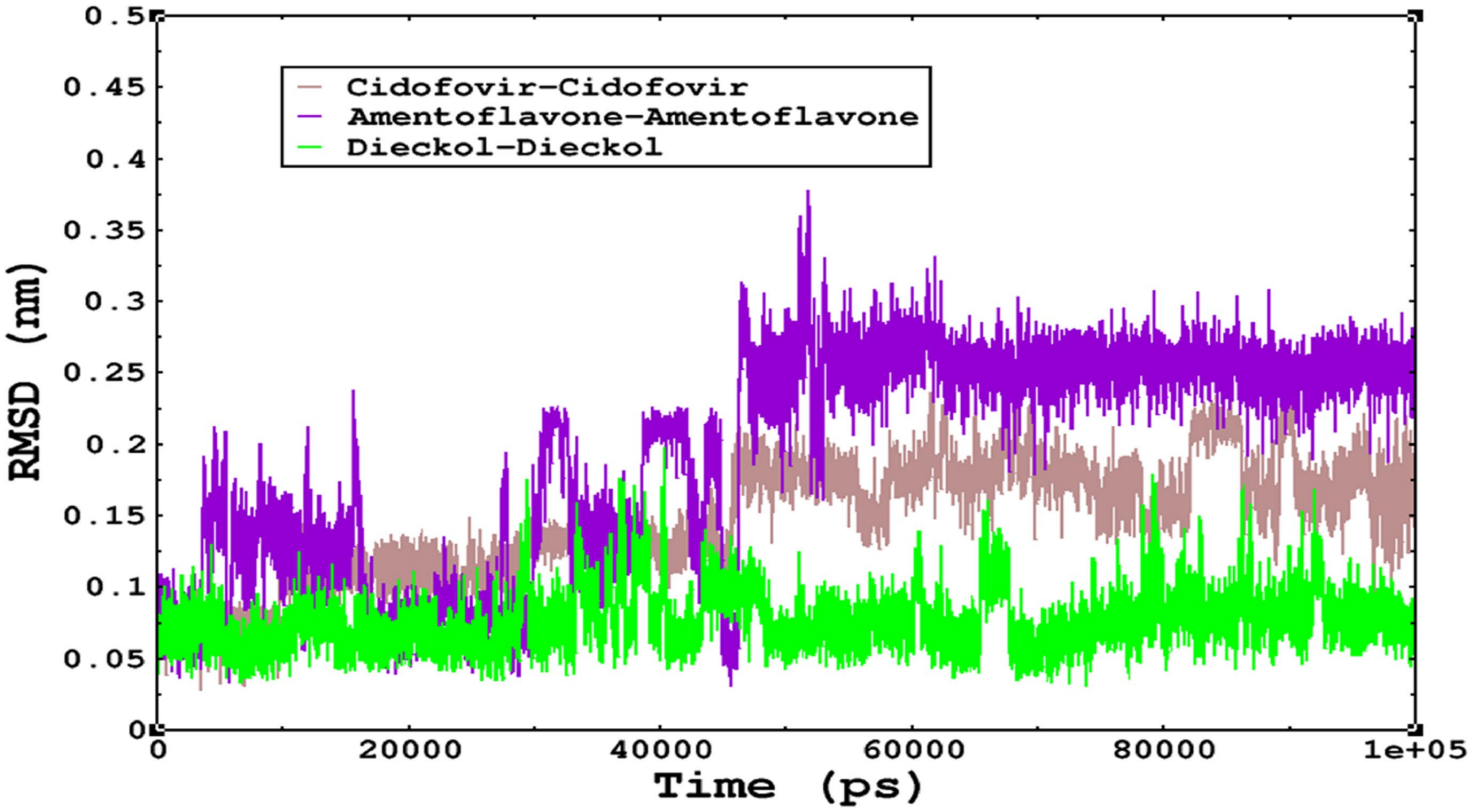
The RMSD of cidofovir, amentoflavone, and dieckol was calculated after superposing on the same molecular scaffold in the aqueous medium for 100 ns of the simulation period.

Molecular dynamics simulations are essential to determining the structural conformations of protein-ligand complexes. Promising interactions of dieckol with PDB *4QWO* further encourage us to perform MD simulations. Figure 4 shows the RMSD of dieckol, amentoflavone, and cidofovir with monkeypox virus profilin-like protein. It was observed that the RMSD of Dieckol remains between 0.1–0.2 nm throughout the simulations, but some jumps of dieckol have also been observed at the time scales of 40, 80, 83, and 85 ns of the simulations; this does not show any effect on the ligand binding with the binding site. Dieckol remained stable in the binding site throughout the simulation period. The RMSD of amentoflavone with monkeypox virus profilin-like protein remained intact throughout the simulation period. Also, the reference ligand, cidofovir, is a common drug used to treat the *monkeypox* virus. The MD simulations revealed that Cidofovir showed robust binding with the *monkeypox* protein for 45 ns of the time scale, then the cidofovir showed a higher change in conformations after 98 ns of the period, then unbinding of the cidofovir was observed after 98 ns of the period. This study demonstrated that dieckol is more stable than cidofovir and amentoflavone, as observed from the conformations of the molecules during simulations. Therefore, dieckol will exert more significant inhibition of the *monkeypox* virus than cidofovir. Next, each ligand was superposed with the respective ligand, and the conformations were calculated and represented in the form of root mean square deviations (RMSD) in Figures 4, 5.

The stability of the complexes was determined from the Cα backbone of the monkeypox virus protein complexed with cidofovir, amentoflavone, and dieckol. The Cα backbone is a crucial parameter in the MD simulation to determine the stability of the complexes. The *monkeypox* virus complexed with cidofovir, amentoflavone, and dieckol was compared with the apo complex. This analysis logically gives an in-depth idea of the effects on the structures due to the ligand binding. The RMSD of the apo complex (without ligand) ranged between 0.1–0.15 nm and reflects the stable trajectory throughout the simulation period parallel to the amentoflavone-bound monkeypox virus. The complex reference cidofovir bound to monkeypox showed minor fluctuations for 10–20 ns, then became stable. Again, minor fluctuations were observed in the RMSD on a time scale of 60–70 ns. Finally, dieckol complexed with the monkeypox virus showed stability throughout the simulation period, and the RMSD was found to be 0.9–1.1 nm. This study shows that dieckol is the most stable and has no effect on the Cα backbone of the *monkeypox* virus.

**Figure 6:**
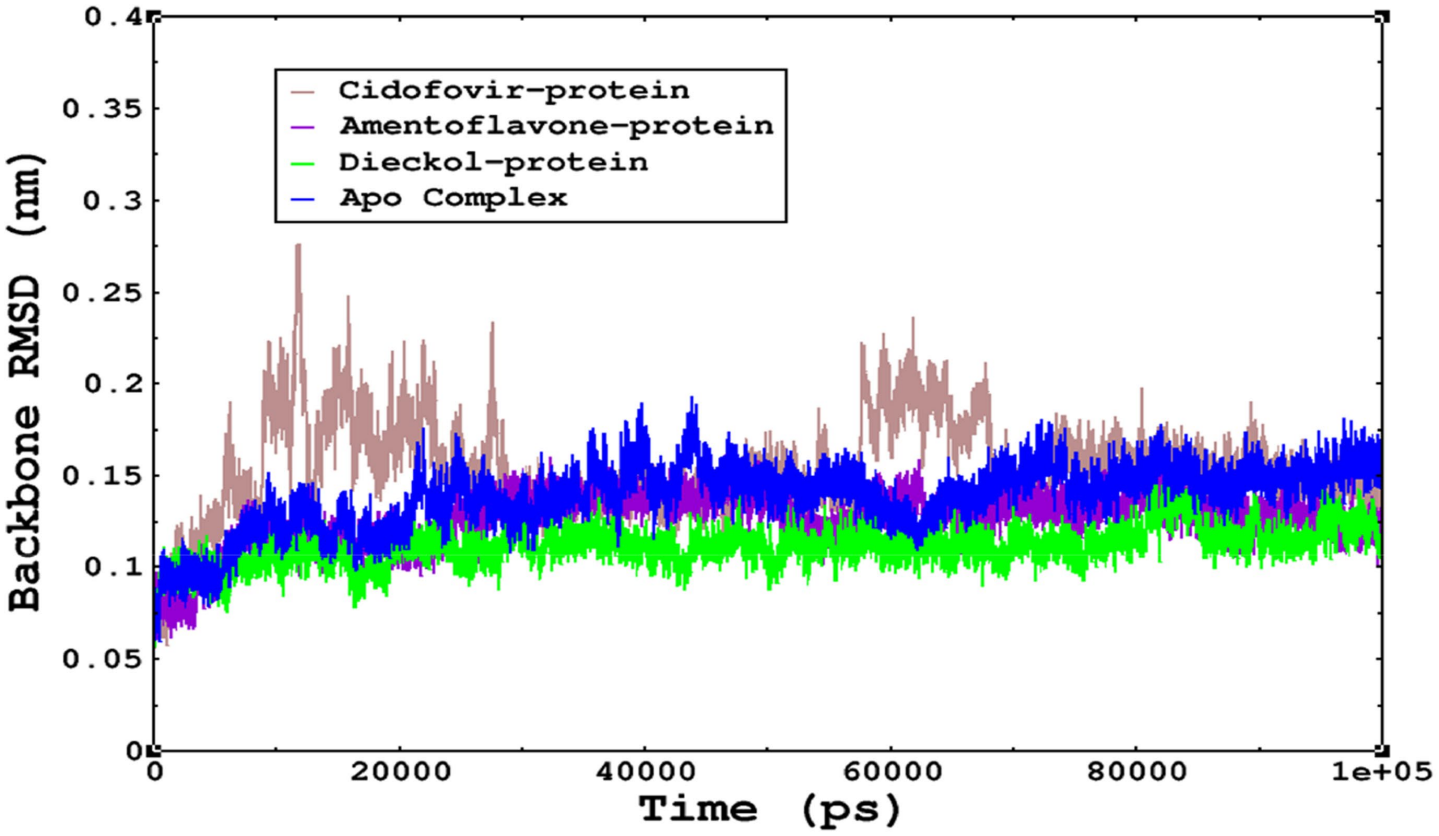
The stability of the complexes was monitored from the Cα backbone during the simulations; all complexes were found to have a stable Cα backbone; the most stable complex is dieckol.

**Figure 7:**
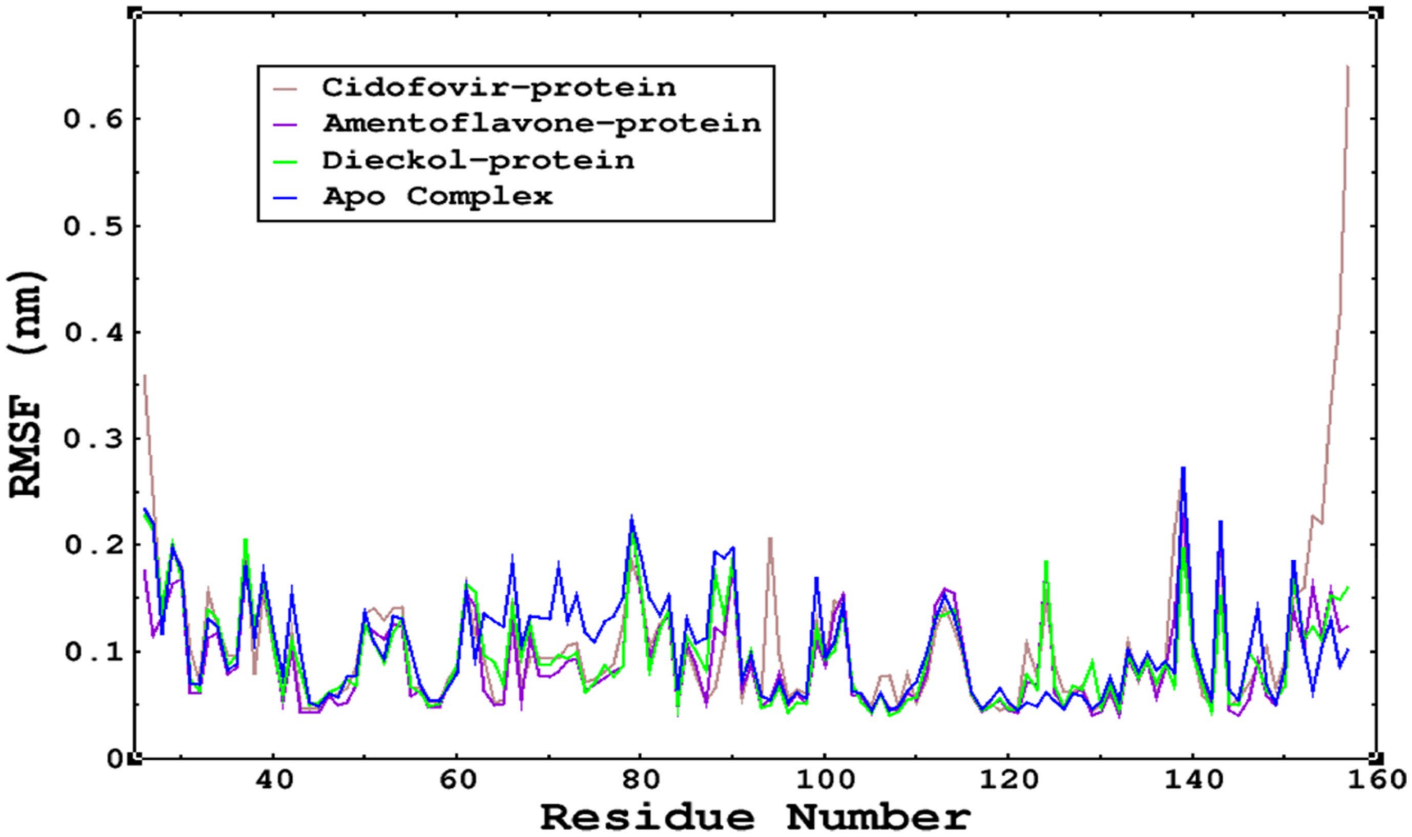
The RMSF was calculated by using the RMSF module of GROMACS; parallel fluctuations were observed between the Apo complex and the other complexes complexed with the ligands. This demonstrates no significant fluctuations between Apo and other complexes, except that the reference cidofovir bound to monkeypox shows higher fluctuations at the terminal end.

The root mean square fluctuations were found to be within an acceptable range; no higher fluctuations were observed; only the reference complex Cidofovir bound to monkeypox showed higher fluctuations at the terminal end. The limited fluctuations reveal the stability of the complexes in Figures 6, 7.

**Figure 8:**
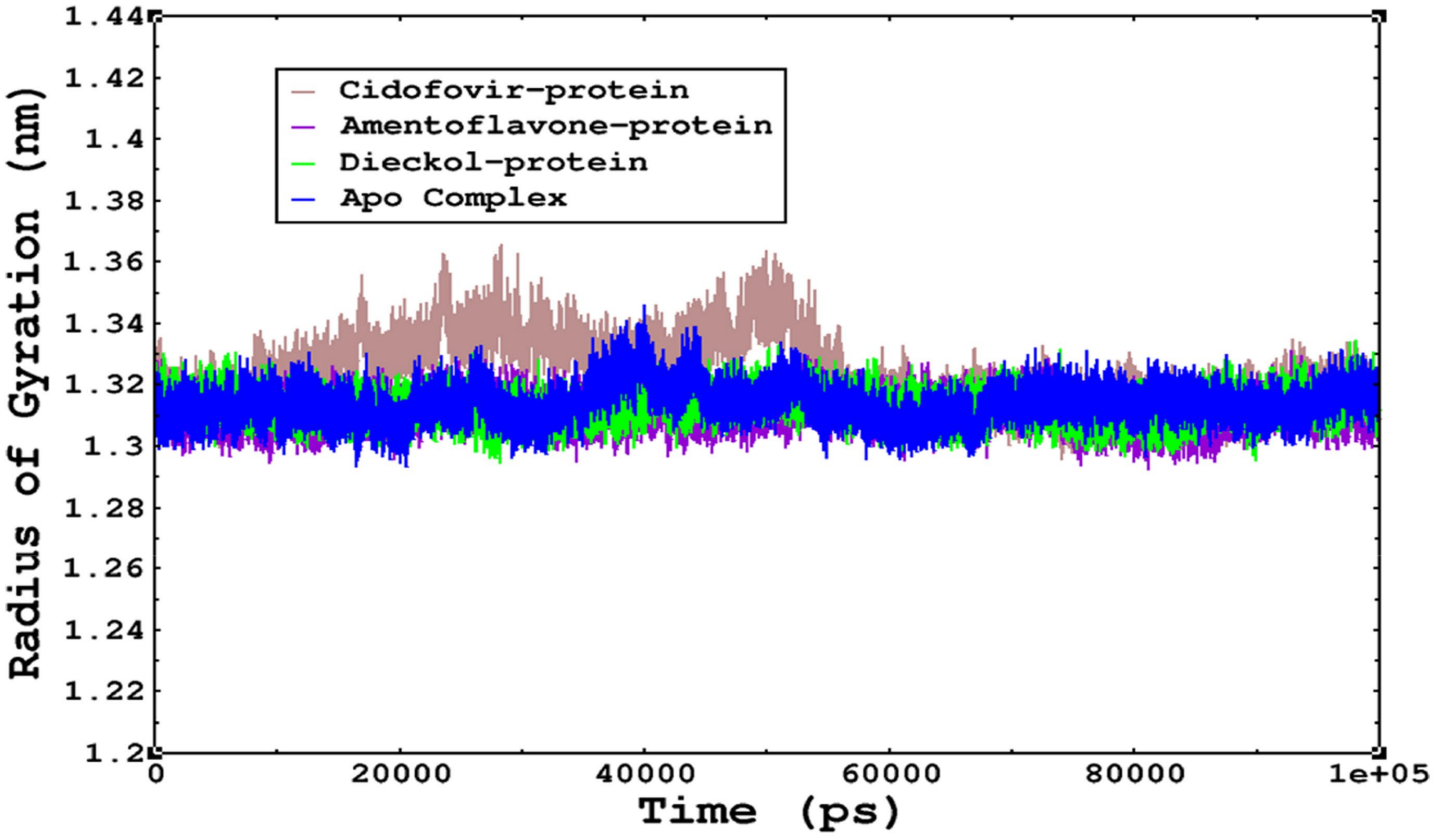
The radius of gyration refers to the compactness and rigidity of the complexes. The ROG trajectories are represented in the legend box for each complex.

**Figure 9:**
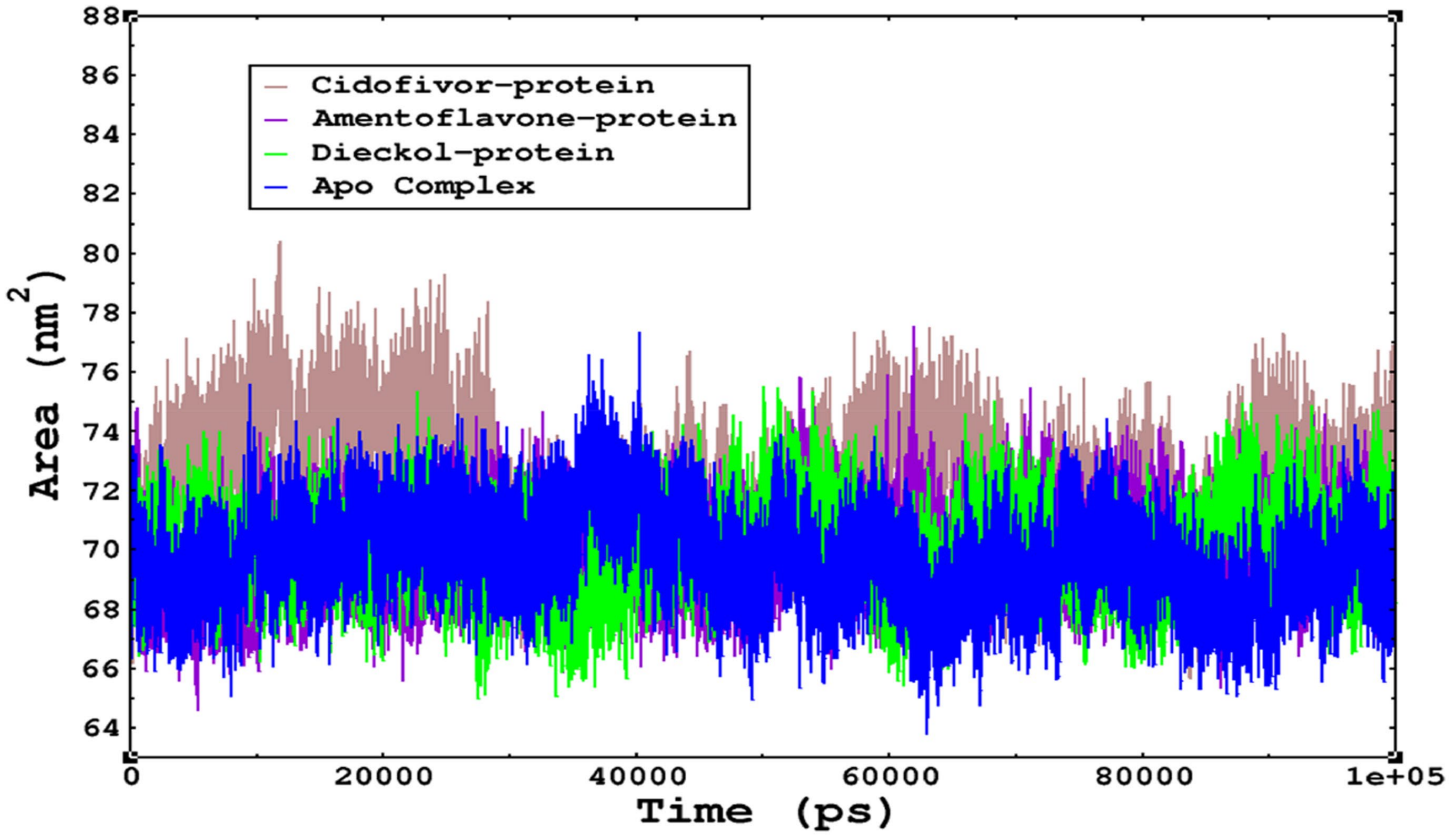
The SASA values of the complexes as calculated by gmx_sasa. The reflected SASA trajectories were plotted for each complex, and the complex representations are given in the legend box.

The compactness of the complexes was monitored, which primarily determines their rigidity. The higher the value of the radius of gyration (ROG), the more unstable the complex (Ghahremanian et al., 2022). Lower values result in greater stability in the complex. The apo complex, amentoflavone, and dieckol bound to the *monkeypox* virus showed ROG values of 1.3–1.33 nm throughout the simulation period, and similar compactness results in the stability of the complex. However, the reference complex Cidofovir bound with monkeypox showed higher ROG until 58 ns of the simulation period and then achieved ROG values that were comparatively similar to those of other complexes in the present study (Figure 8). Protein folding was calculated from the solvent-accessible surface area (SASA). In the reference complex, the SASA value is comparatively higher than in the other complexes in the present study. Similarly, the lower SASA value demonstrates a higher protein folding and is referred to as a stable complex, whereas the higher protein folding results from the instability of the complexes. These results demonstrated that dieckol bound to the *monkeypox* virus showed linear trajectories and was correlated with the ROG (Figure 9).

#### 3.7.1. Secondary structure analysis of proteins

**Figure 10:**
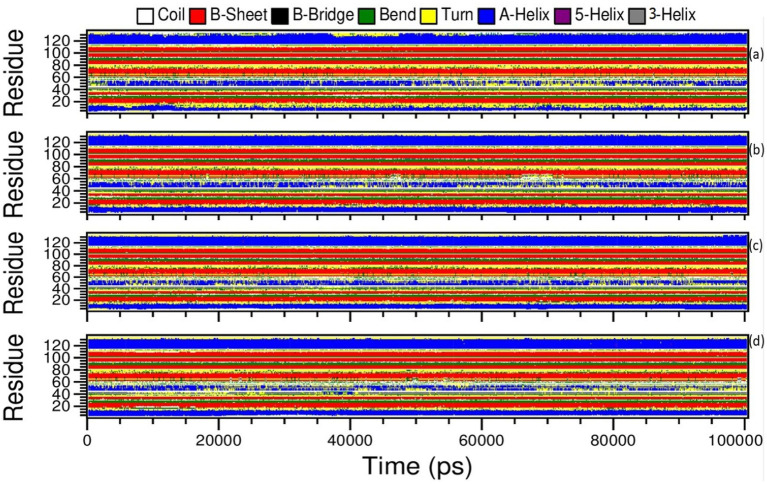
Definition Secondary Structure of Protein (DSSP) analysis of Amentoflavone, Dieckol, Cidofovir bound monkeypox, and Apo complex.

The Definition Secondary Structure of Proteins (dssp) module in GROMACS was chosen to determine the structural changes of the *monkeypox* virus upon binding to amentoflavone and dieckol. The structural changes were correlated with the apo complex and the reference complex of cidofovir-bound *monkeypox*. Moreover, the changes in the apo complex and the reference complex (cidofovir-bound *monkeypox* protein) were observed from the dssp plot. The amino acids located between 44–56 ns showed transitions between α-helix and loop turns at position 55 ns of the time scale until the end of the simulations (Figure 10A).

Similarly, the reference complex showed bend changes into the loop turns, and the transitions occur among amino acids residing 44–56, and transitions occur between α-helix, and loop turns at 20, 41, 45, 70–80, and 90–100 ns of time scale during simulations (Figure 10D).

In the complex amentoflavone and dieckol bound to the monkeypox virus, the minor transitions occurred at amino acids residing at 44–56 ns, and transitions occurred between α-helix and loop turns at varied simulation periods (Figures 10B,C). The overall understanding of the simulations is that amentoflavone and dieckol bound to the monkeypox virus are stable complexes and need further investigation.

#### 3.7.2. Free binding energy calculations

The free binding energy was calculated using the MM\PBSA method. The 60–100 ns generated trajectory was used for the free binding energy calculations (Table 6). All the molecules included in the present study showed lower contributions to hydrogen bonding. At the same time, a hydrophobic interaction was dominant in each complex. The binding energy exhibited by amentoflavone was found to be −115.610 ± 1.531 kJ/mol, and for the dieckol complex, the binding energy was −207.080 ± 1.797 kJ/mol. The reference complex cidofovir exhibited ΔG_bind_ 212.357 ± 2.766 kJ/mol, and the positive ΔG_bind_ obtained by cidofovir was due to the unbinding that occurred during the simulations.

**Table 6:** Free binding energy data.

Ligand	ΔE_vdw_ kJ/mol	ΔE_Elec_ kJ/mol	ΔE_Polar solvation_ kJ/mol	SASA kJ/mol	ΔE_binding Energy_ kJ/mol
Amentoflavone	−168.239 ± 0.535	−20.276 ± 1.077	80.607 ± 1.282	−14.709 ± 0.038	−115.610 ± 1.531
Dieckol	−197.578 ± 1.083	−215.037 ± 2.576	226.411 ± 1.348	−20.845 ± 0.075	−207.080 ± 1.797
Cidofovir	−66.350 ± 0.922	250.383 ± 4.145	37.585 ± 2.008	−9.241 ± 0.094	212.357 ± 2.766

### 3.8. *Babesia microti* complexes MD simulations

**Figure 11:**
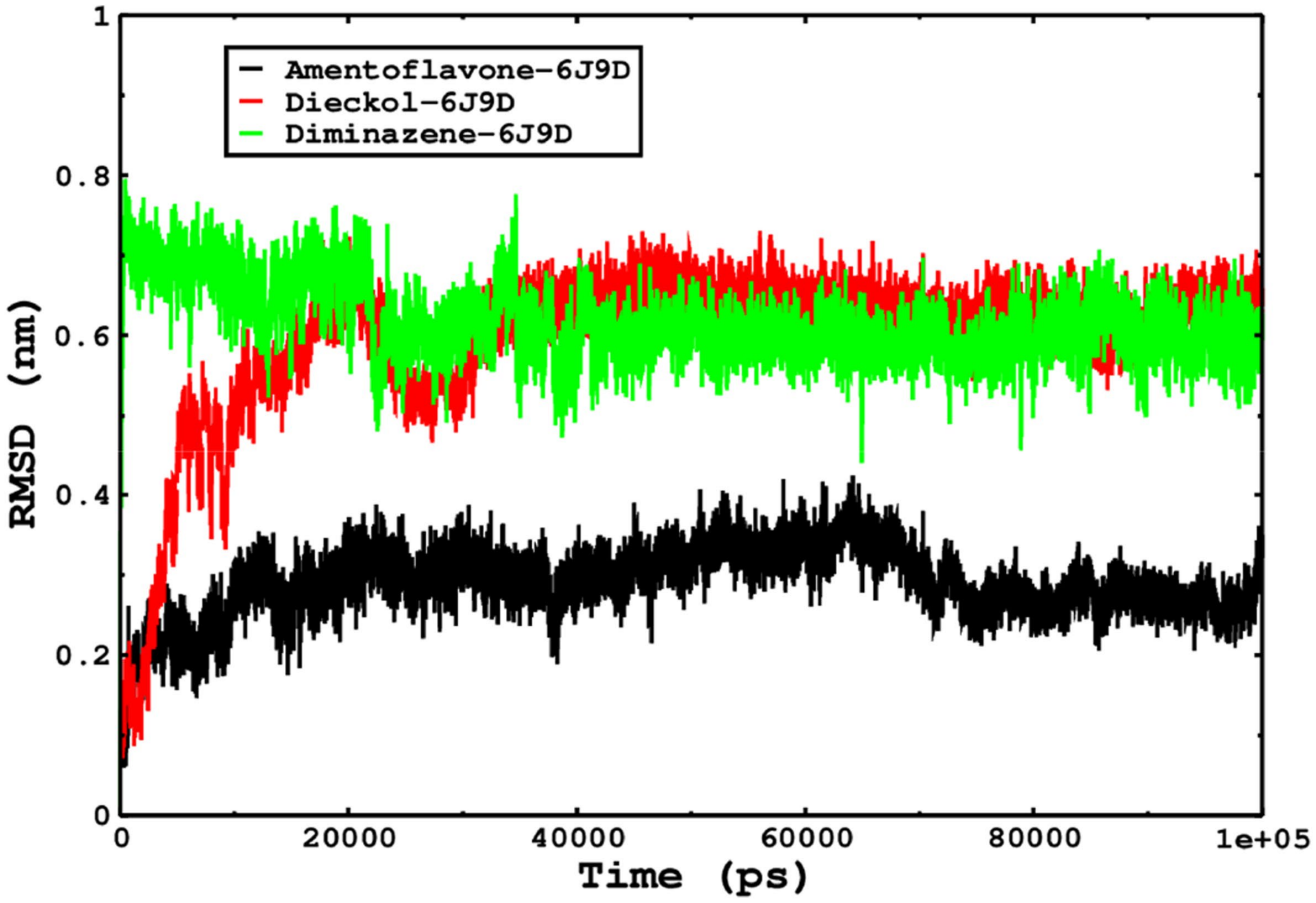
The ligand conformations were monitored in the aqueous medium for 100 ns of simulations. The RMSD of each ligand was monitored by superposition on the protein 6J9D, and the plotted trajectories are represented in the legend box.

**Figure 12:**
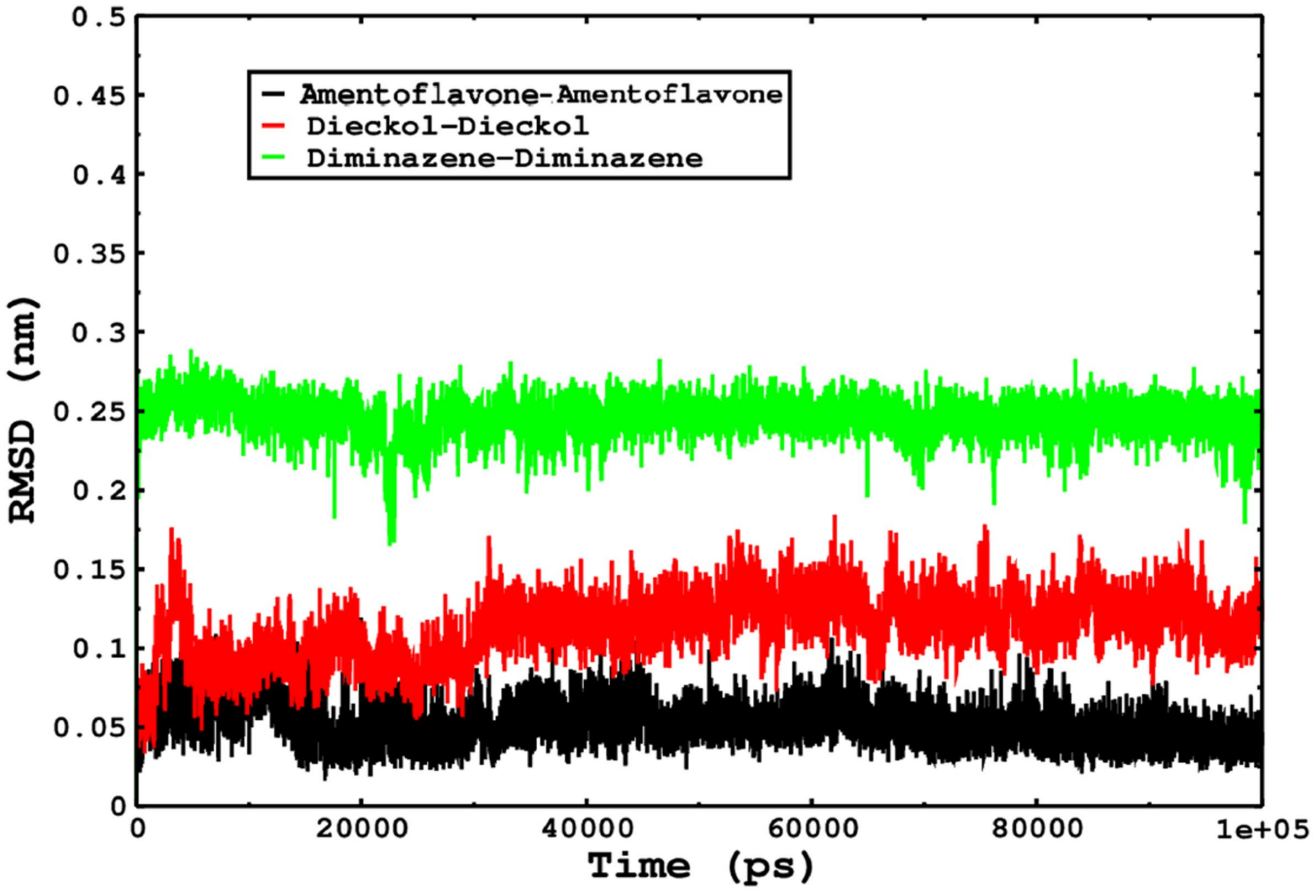
The conformations of only ligands were monitored by superposition on the respective ligand, and the conformations were plotted in the form of RMSD.

**Figure 13:**
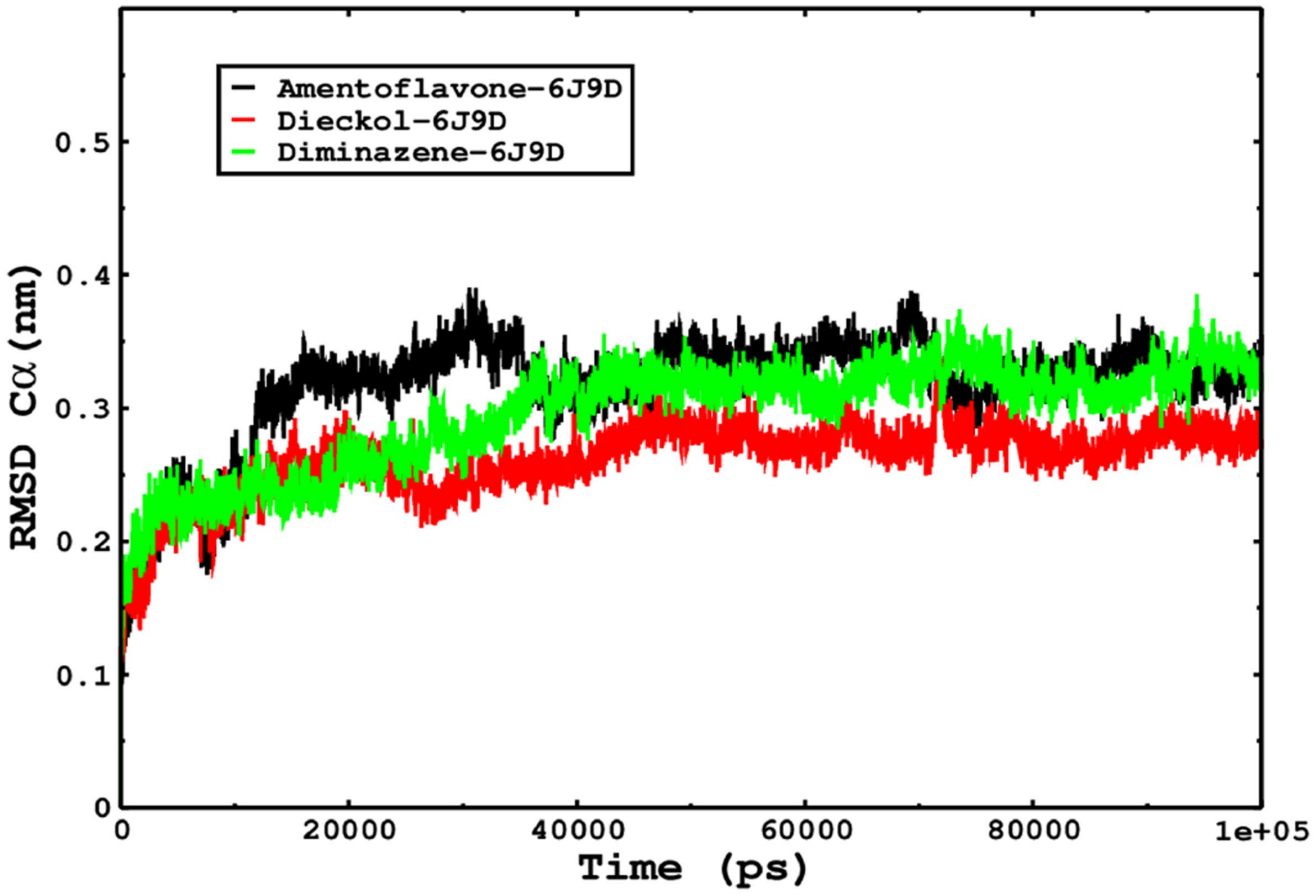
The protein stability of each complex simulated for 100 ns was determined from the conformations of the Cα, and the RMSD Cα indicates that each complex was found stable.

**Figure 14:**
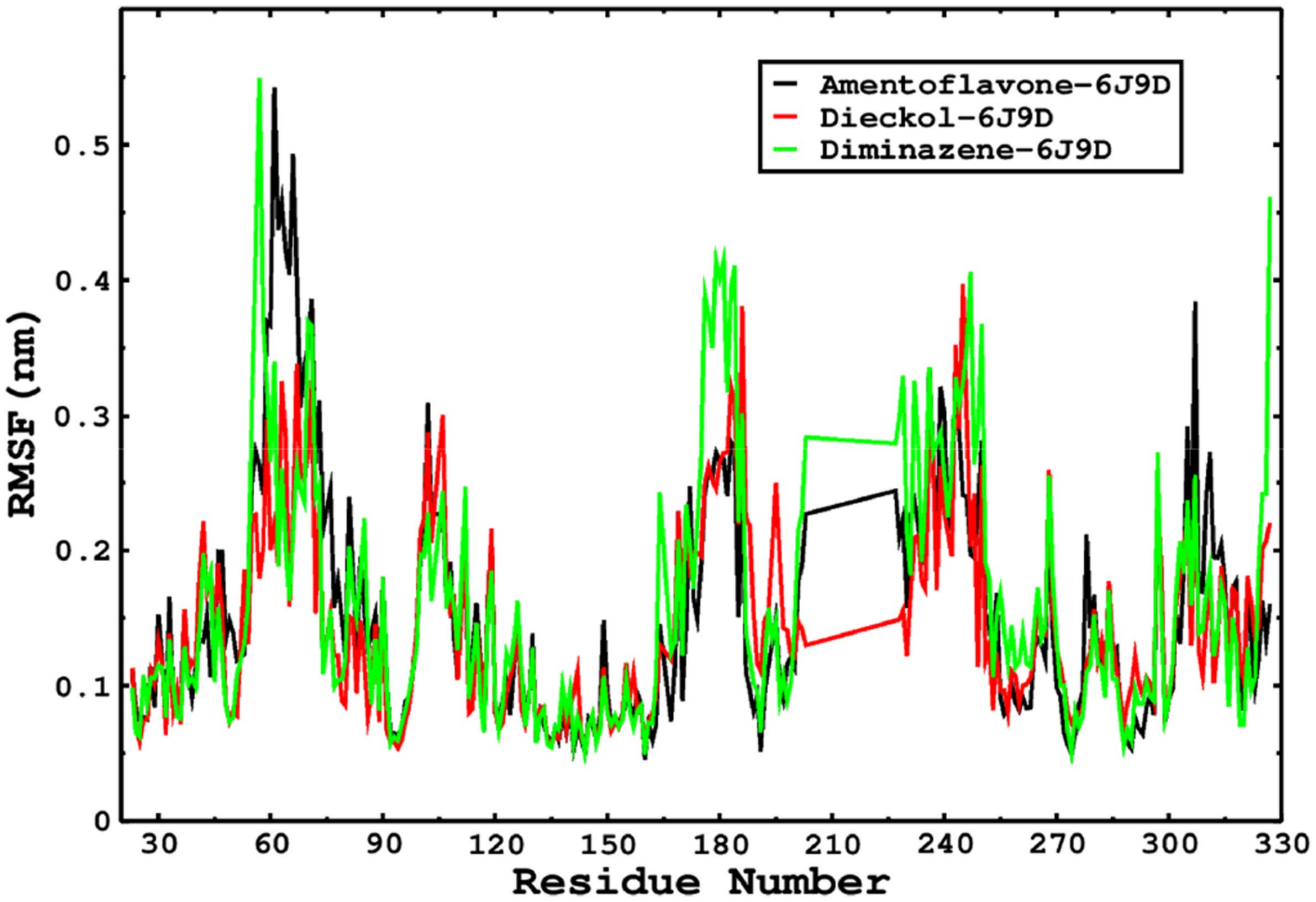
The RMSF was calculated by using the RMSF module of the GROMACS; the parallel fluctuations complexed with the ligands were found in parallel.

**Figure 15:**
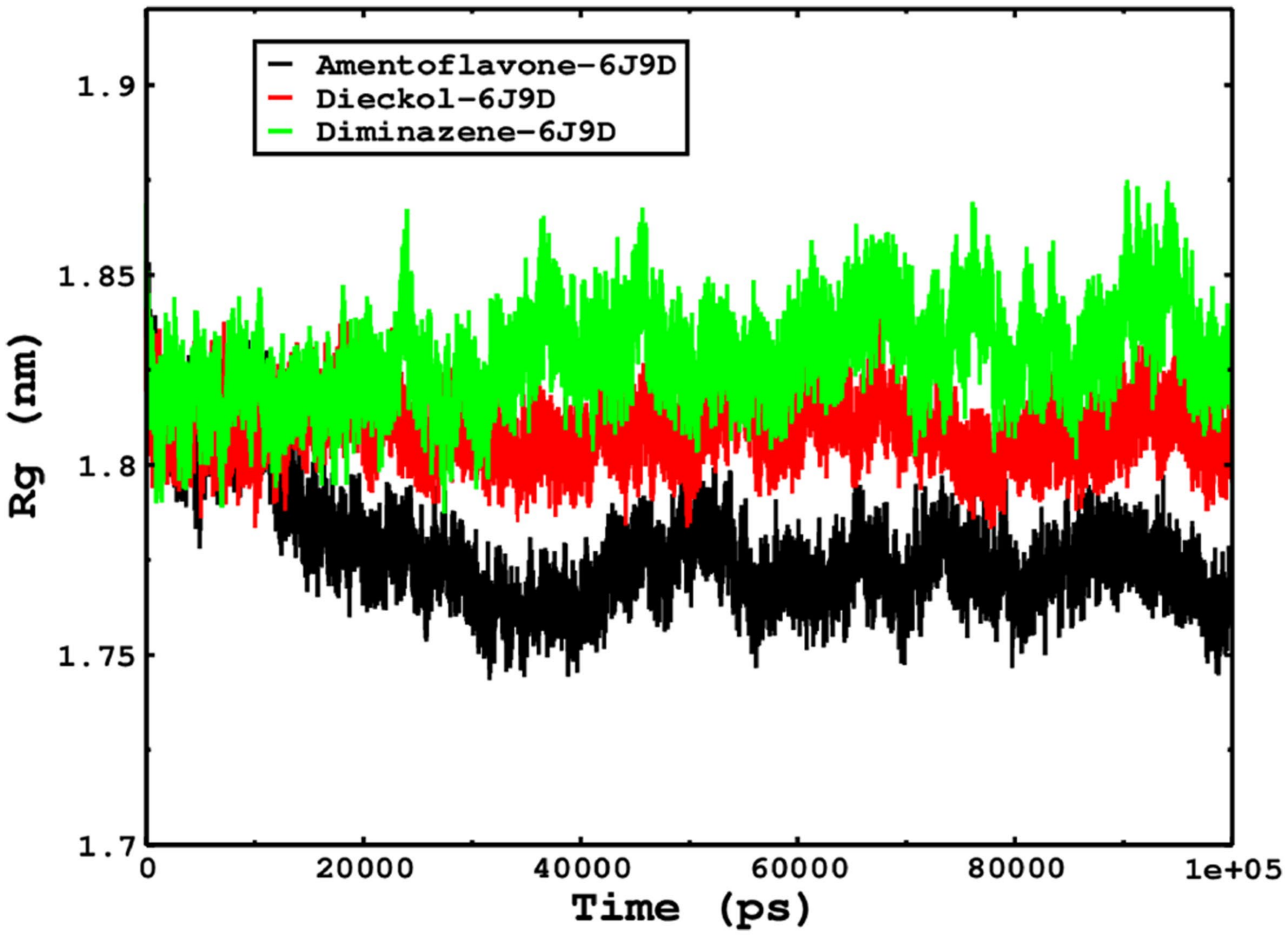
The radius of gyration is related to the compactness and rigidity of the complexes. The ROG trajectories are shown in the legend box for each complex.

**Figure 16:**
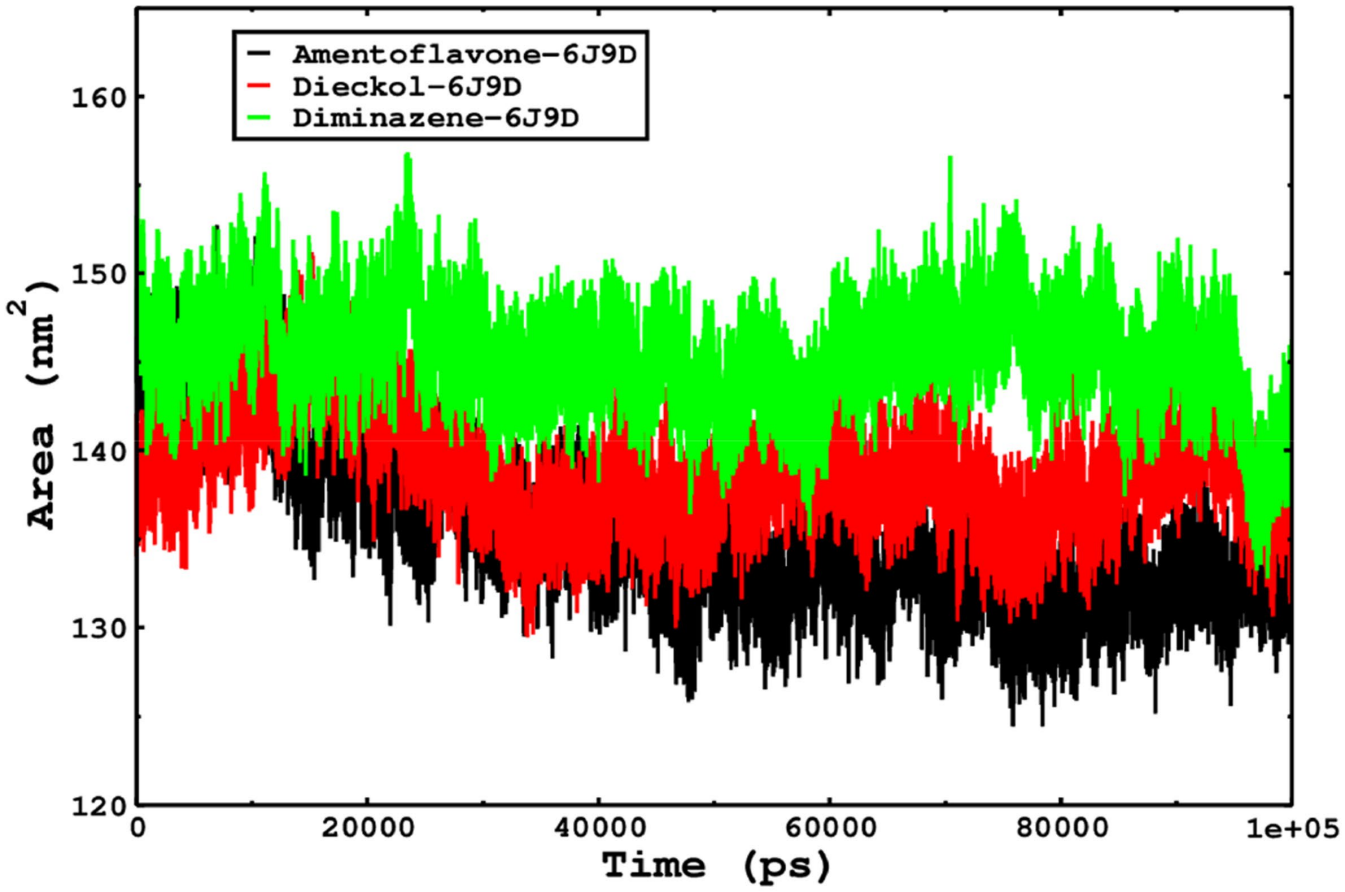
The SASA of the complexes was calculated by gmx_sasa. The reflected SASA trajectories were plotted for each complex, and the complex representations are shown in the legend box.

We also conducted molecular dynamics simulations of amentoflavone, dieckol, and diminazene complexed with *Babesia microti* lactate dehydrogenase (PDB ID 6J9D). The RMSD of each ligand was monitored. It was found that each ligand was stabilized in the binding site, and the orientations were represented in the form of RMSD. It was observed that the RMSD of amentoflavone ranged between 0.2–0.38 nm throughout the simulations. The RMSD of dieckol with protein (PDB 6J9D) remained intact with the protein throughout the simulation period. Moreover, the standard ligand diminazene MD simulations revealed binding with the *Babesia microti* lactate dehydrogenase protein for 100 ns of the time scale, and the conformations showed stability from the start to the end of the simulations. This study demonstrated that amentoflavone, dieckol, and diminazene were found to be stable, as observed from the conformations of the molecules during simulations. Therefore, amentoflavone, dieckol, and diminazene will significantly inhibit *Babesia microti* lactate dehydrogenase. Next, each ligand was superposed with the respective ligand, and the conformations were calculated in the form of RMSDs (Figures 11, 12). The stability of the whole protein was determined by calculating the RMSD Cα. The backbone Cα revealed that each complex remained stable throughout the simulation period. The conformations of backbone Cα were calculated in the form of RMSD Cα and the data points were found to be parallel to each other. No major changes in RMSD were observed, with the RMSD Cα ranging between 0.2–0.4 nm throughout the simulation period (Figure 13). The root mean square fluctuations of each amino acid present in the *Babesia microti* lactate dehydrogenase (PDB ID 6J9D) complexed with amentoflavone, dieckol, and diminazene were investigated during the simulation period. Fewer fluctuations indicated protein stability. The binding site amino acids showed fewer fluctuations, confirming that the binding of amentoflavone, dieckol, and diminazene does not affect the protein stability (Figure 14). Moreover, the compactness of each protein was investigated by calculating the radius of gyration; higher values result in protein instability, whereas lower values of the radius of gyration result in a more compact protein structure. The protein complexed with amentoflavone exhibited a lower radius of gyration value than the other two complexes, i.e., 6J9D complexed with dieckol and diminazene (Figure 15). Next, the protein folding was determined by calculating the solvent-accessible surface area. The higher the protein folding, the higher the SASA values. From the SASA plots, it was found that amentoflavone, dieckol, and diminazene complexed with 6J9D were parallel, and the same protein folding was observed (Figure 16). Overall, these findings show that each complex of amentoflavone, dieckol, and diminazene complexed with *Babesia microti* lactate dehydrogenase was found to be stable. Further, *in vitro* experiments are required to validate this computational study.

#### 3.8.1. Free binding energy calculations of the *Babesia microti* lactate dehydrogenase complex

The free binding energy was calculated using the MM\PBSA method. The 60–100 ns generated trajectory was used for the free binding energy calculations (Table 7). All the molecules included in the present study showed lower contributions to hydrogen bonding. At the same time, the hydrophobic interaction was dominant in each complex. The binding energy exhibited by amentoflavone was found to be −220.278 ± 2.302 kJ/mol, and for the dieckol complex, the binding energy was −252.554 ± 3.043 kJ/mol. The reference complex cidofovir had a ΔG_bind_ of −38.166 ± 6.111 kJ/mol; the positive ΔG_bind_ obtained by cidofovir was due to the unbinding that occurred during the simulations.

**Table 7:** Free binding energy data.

Ligand	ΔE_vdw_ kJ/mol	ΔE_Elec_ kJ/mol	ΔE_Polar solvation_ kJ/mol	SASA kJ/mol	ΔE_binding Energy_ kJ/mol
Amentoflavone	−245.713 ± 1.353	−94.143 ± 2.019	141.932 ± 1.144	−22.344 ± 0.122	−220.278 ± 2.302
Dieckol	−269.596 ± 1.589	−162.381 ± 3.689	210.245 ± 1.678	−30.788 ± 0.118	−252.554 ± 3.043
Diminazene	−126.488 ± 1.637	−247.210 ± 10.249	352.501 ± 4.680	−17.069 ± 0.128	−38.166 ± 6.111

### 3.9. Frontier molecular orbitals and chemical reactivity descriptors

The following descriptors were used to determine the behavior of the phytochemicals selected in this study. The energy band gap between HOMO and LUMO was calculated, from which the Chemical Reactivity Descriptors were determined. This parameter is essential to determining the stability of the molecule. A higher band gap between HOMO and LUMO results in a more stable molecule, whereas a lower energy gap exhibits higher reactivity and is a useful parameter in determining kinetic stability. The energy gap was found to range between 3 and 6 eV, which indicates that the chosen molecules are stable and reactive (Table 8). The HOMO and LUMO data calculations and frontier molecular orbital images are represented in Table 7; Supplementary Figure S1. The stability order of the molecules is as follows: alloaromadendrene, isofucosterol, dieckol, tomentin a, rhoifolin, amentoflavone, psoralidin, and pectolinarin. Their reactivity is in the reverse order of the above-represented molecules. Hardness determines how hard a compound is, and softness determines how quickly a compound dissolves or breaks down when in contact with the liquid phase. Hardness and softness are opposites of each other, and it is seen that the softness value for all compounds is much lower in comparison to hardness. Thus, it is estimated that these compounds will dissolve quickly (Rahman et al., 2022).

**Table 8:** Chemical reactivity descriptors.

Drug Name	LUMO	HOMO	Gap	I = -HOMO	A = -LUMO	Hardness	Softness
Alloaromadendrene	−0.3235	−6.5184	6.1949	6.5184	0.3235	3.4209	0.3228
Isofucosterol	−0.3899	−6.3731	5.9832	6.3731	0.3899	2.9916	0.3343
Amentoflavone	−2.4852	−6.4969	4.0118	6.4969	2.4852	2.0059	0.4956
Psoralidin	−2.0989	−6.0303	3.9314	6.0303	2.0989	1.9656	0.5088
Dieckol	−0.5659	−4.8836	4.3176	4.8836	0.5659	2.1599	0.4632
Tomentin A	−2.0173	−6.3302	4.2784	6.3302	2.0173	2.1394	0.4675
Pectolinarin	−2.2677	−5.9919	3.7242	5.9919	2.2677	1.8620	0.5370
Rhoifolin	−2.5518	−6.6515	4.0996	6.6515	2.5518	2.0498	0.4878

## 4. Discussion

The monkeypox virus and the *Babesia microti* parasite are highly pathogenic infectious diseases that have emerged as a growing public health concern in recent years. There is currently no specific treatment or vaccine available for these diseases, and they can cause severe illness and even death in some cases. There are significant differences between these two pathogens in their biology, physiological characteristics, and pathogenesis. The primary objective of this investigation is to inhibit the monkeypox virus profilin-like protein and *Babesia microti* lactate dehydrogenase. If this is possible, it would likely prevent and limit their ability to cause disease. In the case of monkeypox, inhibiting this target protein would likely prevent the virus from replicating in infected cells, reducing the severity of the infection and potentially preventing the spread of the virus to other individuals. On the other hand, inhibiting the target protein of *Babesia microti* would prevent the parasite from multiplying inside red blood cells, which is essential for its survival and ability to cause disease. This would likely limit the severity of the infection and reduce the risk of complications such as anemia that can result from the destruction of infected red blood cells.

Second, nature is an excellent source for novel drug development. So, the mentioned phytochemicals have been selected for having many pharmacological activities against different infectious diseases, and based on the literature, this study has been designed to investigate the dual function of some of these compounds against the mentioned virus and parasite using advanced computational and drug design approaches such as molecular docking, molecular dynamics simulation, DFT, ADMET, drug-likeness, etc. The molecular docking score was reported as outstanding against both pathogens. Then, free binding energy, molecular dynamics simulation, DFT, and other related studies are performed step by step, and all the levels of computational experiments are satisfied by the mentioned phytochemicals. Finally, this section should conclude that these phytocompounds could be further studied in a wet lab to investigate their actual performance and validate the computational result.

## 5. Conclusion

The application of advanced computational strategies and combined drug design approaches, such as ADMET evaluation, ligand drug-likeness quantification, and molecular docking analysis, has led to the identification and characterization of potential inhibitors of the viral pathogens *Babesia microti* and monkeypox. Through these methods, a total of 11 promising lead compounds, including Alloaromadendrene, Isofucosterol, Amentoflavone, Psoralidin, Dieckol, Tomentin A, Chlorogenic acid, Pectolinarin, Rhoifolin, Oxymatrine, and Sanguinarine, have been demonstrated to have high potency against the active catalytic sites of the target enzymes, outstanding drug-like properties, and no toxic effects. Moreover, the binding affinities of the selected natural biomolecules were measured using the AutoDock Vina tool, resulting in ranges of −6.5 kcal/mol to −11.1 kcal/mol against *Babesia microti* lactate dehydrogenase (PDB ID 6J9D), −6.2 kcal/mol to −10.4 kcal/mol against *Babesia microti* lactate dehydrogenase apo form (PDB ID 6 K12), −6.3 kcal/mol to −10.1 kcal/mol for monkeypox virus profilin-like protein (PDB ID 4QWO), and − 6.9 kcal/mol to −10.5 kcal/mol for monkeypox virus DNA polymerase (PDB ID 8HG1). Notably, Dieckol and Amentoflavone exhibited higher reactivity and better affinity for both the *Babesia microti* and monkeypox-targeted proteins, with high predicted affinities. Dieckol, in particular, demonstrated effective and potent binding ability against monkeypox and remained stable within the binding site during MD simulations. The MM/PBSA method calculated the highest negative free energy, with Amentoflavone and Dieckol showing free binding energies of −115.610 ± 1.531 kJ/mol and − 207.080 ± 1.797 kJ/mol, respectively, while Cidofovir showed a free binding energy of 212.357 ± 2.766 kJ/mol. This research focuses on the inhibition of monkeypox and *Babesia microti* using phytocompounds, and among them, the multifaceted role of Dieckol and Amentoflavone has been discovered, as they surprisingly bind and suppress both monkeypox and *Babesia microti* pathogens effectively.

## 6. Prospects of the study

In conclusion, the prospects of this study include conducting *in vitro* and *in vivo* validation of the lead compounds, particularly Dieckol and Amentoflavone, to assess their efficacy against monkeypox and *Babesia microti*. Structural optimization and combination therapy approaches hold promise for enhancing their potency and broadening their spectrum of activity. Additionally, clinical trials may be conducted to evaluate the safety and effectiveness of these compounds in humans. These future directions will contribute to advancing our understanding and potential treatments for inhibiting monkeypox and *Babesia microti* infections.

## 7. Limitations of the study

Despite the significant findings and promising results obtained in this study, several limitations should be acknowledged. First, it is important to note that this investigation is purely theoretical in nature, relying on computational methods and simulations. While these approaches provide valuable insights and predictions, further validation through extensive *in vitro* and *in vivo* experiments is required. The practical value of these phytochemicals can only be determined through extensive preclinical and clinical studies. Additionally, this work focused on a specific set of target proteins associated with *Babesia microti* and monkeypox, and further investigation should expand the scope to include a broader range of potential targets. Furthermore, the study primarily explored the binding affinities and drug-like properties of the identified lead compounds, but factors such as pharmacokinetics, bioavailability, and potential side effects should be thoroughly investigated to ensure the development of newer and safer drugs from natural sources. Therefore, to fully validate the findings of this investigation and unlock the potential therapeutic applications of these compounds, it is imperative to conduct comprehensive experimental studies that include computational, preclinical, and clinical trials.

## Data availability statement

The raw data supporting the conclusions of this article will be made available by the authors, without undue reservation.

## Author contributions

SA, SAM, NM, and SH: conceptualization. SA, SM, SAM, and NM: methodology. SA and SH: validation. SA, SH, SM, NM, and BN: formal analysis. H-AN, YB, AM, MB, and SA: data curation. SA, H-AN, YB, AM, MB, and SAM: writing—original draft preparation. BN: writing—review and editing and supervision. All authors have contributed to the article and approved the submitted version.

## Conflict of interest

The authors declare that the research was conducted in the absence of any commercial or financial relationships that could be construed as a potential conflict of interest.

## Publisher’s note

All claims expressed in this article are solely those of the authors and do not necessarily represent those of their affiliated organizations, or those of the publisher, the editors and the reviewers. Any product that may be evaluated in this article, or claim that may be made by its manufacturer, is not guaranteed or endorsed by the publisher.
