# Supplementary material for: Novel computational and drug design strategies for inhibition of monkeypox virus and Babesia microti: molecular docking, molecular dynamic simulation and drug design approach by natural compounds
Source: Front Microbiol. 2023 Jul 19;14:1206816. doi: 10.3389/fmicb.2023.1206816 (PMC10394520; doi:10.3389/fmicb.2023.1206816)
Supplement: Supplementary file 1 [file Table_1.DOCX]

**Supplementary Figure: S1**

| **Title** | Alloaromadendrene | | Isofucosterol | Amentoflavone |
| --- | --- | --- | --- | --- |
| **LUMO** | 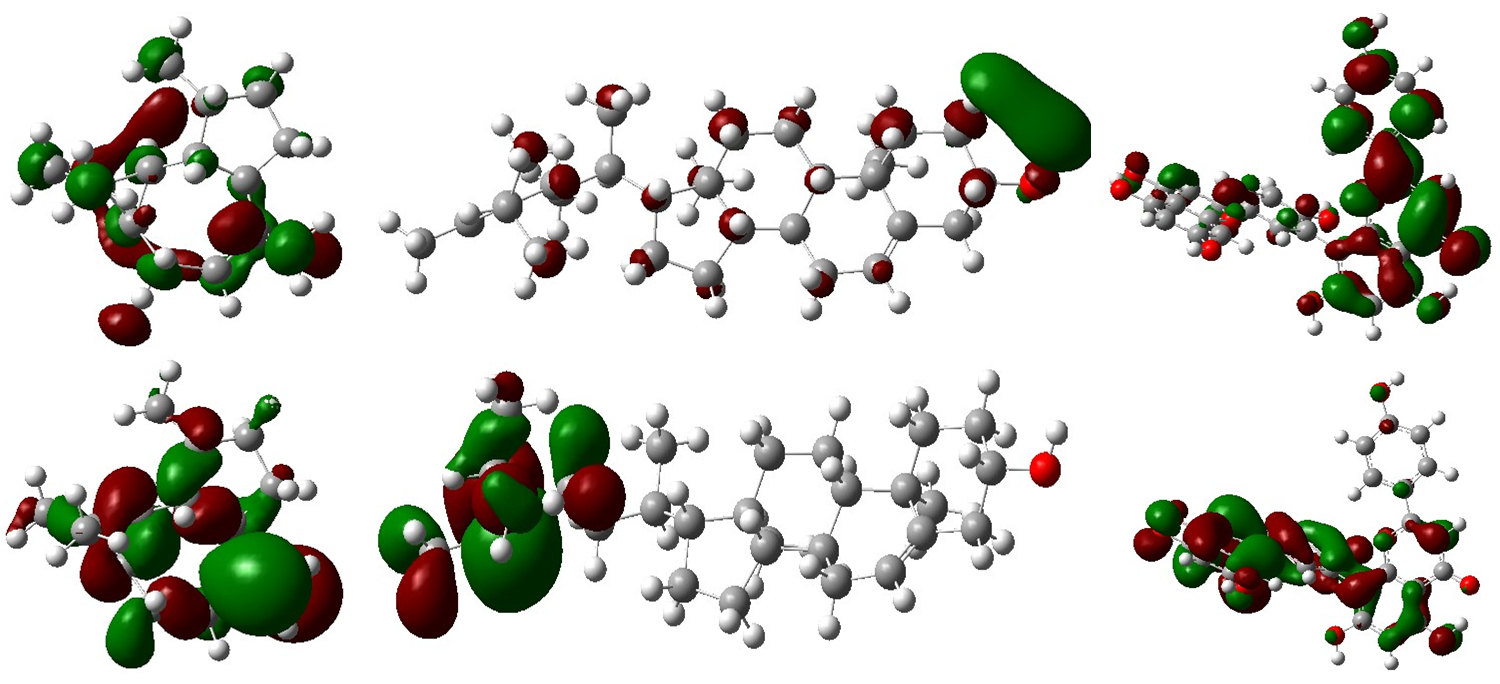 | | | |
| **HOMO** |  |  |  |  |
|  | Psoralidin | Dieckol | | Tomentin A |
| **LUMO** | 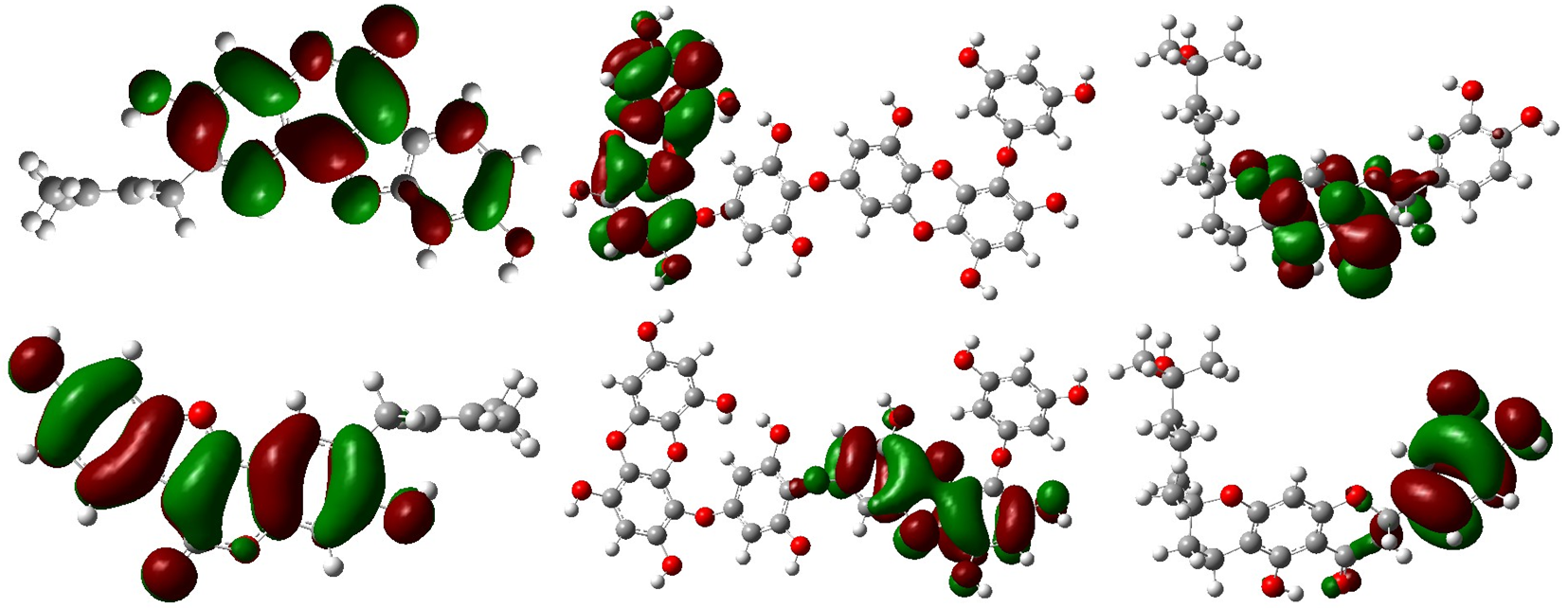 | | | |
| **HOMO** |  |  |  |  |
|  | Pectolinarin Rhoifolin | | | |
| **LUMO** | **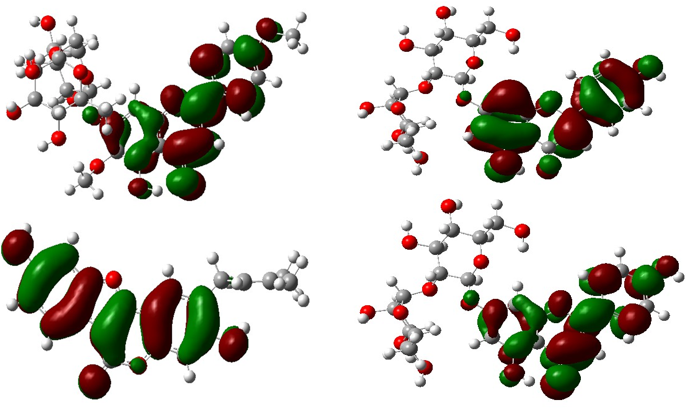** | | | |
| **HOMO** |  |  |  |  |
